# Shooting at Moving and Hidden Targets—Tumour Cell Plasticity and the Notch Signalling Pathway in Head and Neck Squamous Cell Carcinomas

**DOI:** 10.3390/cancers13246219

**Published:** 2021-12-10

**Authors:** Joanna Kałafut, Arkadiusz Czerwonka, Alinda Anameriç, Alicja Przybyszewska-Podstawka, Julia O. Misiorek, Adolfo Rivero-Müller, Matthias Nees

**Affiliations:** 1 Department of Biochemistry and Molecular Biology, Medical University of Lublin, ul. Chodzki 1, 20-093 Lublin, Poland; joannakalafut@umlub.pl (J.K.); arkadiuszczerwonka@umlub.pl (A.C.); 62005@student.umlub.pl (A.A.); alicja.przybyszewska@umlub.pl (A.P.-P.); adolfo.rivero-muller@umlub.pl (A.R.-M.); 2 Department of Molecular Neurooncology, Institute of Bioorganic Chemistry Polish Academy of Sciences, ul. Noskowskiego 12/14, 61-704 Poznan, Poland; jmisiorek@man.poznan.pl; 3 Western Finland Cancer Centre (FICAN West), Institute of Biomedicine, University of Turku, 20101 Turku, Finland

**Keywords:** head and neck squamous cell carcinoma, HNSCC, Notch signalling pathway, angiogenesis, epithelial-to-mesenchymal transition (EMT), intra-tumour heterogeneity (ITH), tumour cell plasticity, tumour microenvironment (TME), extracellular matrix (ECM), cancer stem cells (CSC), metastasis, human papillomavirus (HPV)

## Abstract

**Simple Summary:**

Cancers in the head and neck region are often aggressive and poorly respond to both irradiation or chemotherapy. Chemotherapy is currently limited by a small number of approved drugs. Newer “targeted” drugs, aiming for specific molecules expressed by tumour cells, have not been as beneficial as expected. Research is now investigating new drug targets, involved in the way how tumour cells interact with non-cancer cells from the stroma, the vasculature, and the immune system within the tumour tissues. These highly dynamic processes assist tumour cells to rapidly adapt to any challenges they may encounter during cancer progression or therapy. One such central molecular mechanism, regulating increased tumour cell plasticity, is the Notch signalling pathway. We currently are only beginning to understand the complex interactions of Notch receptors with their ligands, in a broad spectrum of tumour and tumour-associated cells, and how such interactions could represent targets for cancer chemotherapy and personalized medicine.

**Abstract:**

Head and Neck Squamous Cell Carcinoma (HNSCC) is often aggressive, with poor response to current therapies in approximately 40–50% of the patients. Current therapies are restricted to operation and irradiation, often combined with a small number of standard-of-care chemotherapeutic drugs, preferentially for advanced tumour patients. Only very recently, newer targeted therapies have entered the clinics, including Cetuximab, which targets the EGF receptor (EGFR), and several immune checkpoint inhibitors targeting the immune receptor PD-1 and its ligand PD-L1. HNSCC tumour tissues are characterized by a high degree of intra-tumour heterogeneity (ITH), and non-genetic alterations that may affect both non-transformed cells, such as cancer-associated fibroblasts (CAFs), and transformed carcinoma cells. This very high degree of heterogeneity likely contributes to acquired drug resistance, tumour dormancy, relapse, and distant or lymph node metastasis. ITH, in turn, is likely promoted by pronounced tumour cell plasticity, which manifests in highly dynamic and reversible phenomena such as of partial or hybrid forms of epithelial-to-mesenchymal transition (EMT), and enhanced tumour stemness. Stemness and tumour cell plasticity are strongly promoted by Notch signalling, which remains poorly understood especially in HNSCC. Here, we aim to elucidate how Notch signal may act both as a tumour suppressor and proto-oncogenic, probably during different stages of tumour cell initiation and progression. Notch signalling also interacts with numerous other signalling pathways, that may also have a decisive impact on tumour cell plasticity, acquired radio/chemoresistance, and metastatic progression of HNSCC. We outline the current stage of research related to Notch signalling, and how this pathway may be intricately interconnected with other, druggable targets and signalling mechanisms in HNSCC.

## 1. Introduction

According to the latest GLOBOCAN data [1], head and neck squamous cell carcinoma (HNSCC) originates within the oral cavity, oropharynx, nasopharynx, larynx, or the hypopharynx, and is the fifth leading cancer type by combined 5-year widespread presence worldwide. Similar squamous cell carcinomas (SCC) are also frequently found in the lung (LSCC), the uterine cervix, the skin, and in the oesophagus. Oesophageal squamous cell carcinomas (ESCC) also share many morphological features with HNSCC (and are included in some of the discussions here), but are markedly distinct from the more frequent oesophageal adenocarcinomas [2,3].

### 1.1. The Mutational Spectrum of Head and Neck Squamous Cell Carcinomas (HNSCC)

**Figure 1:**
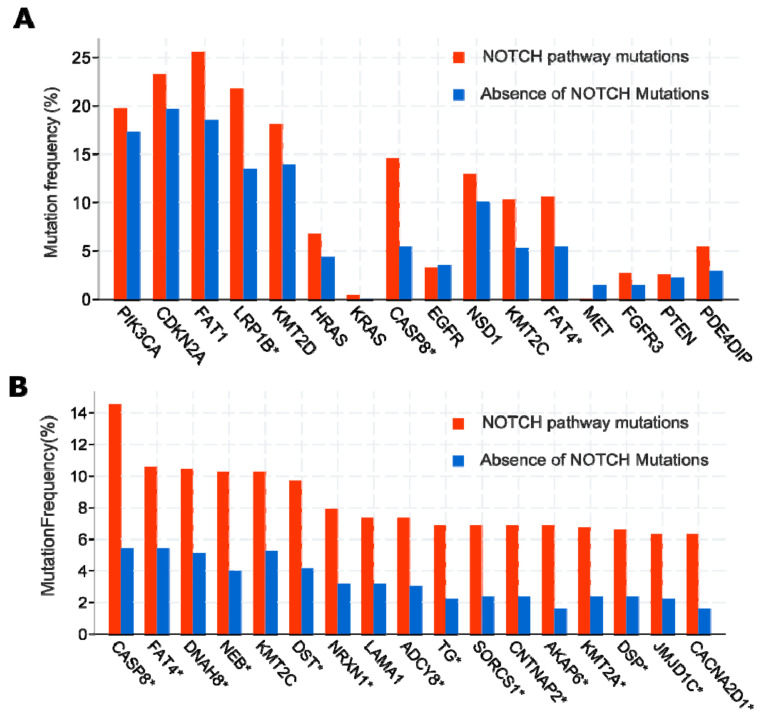
Differential mutation frequency observed in 1332 HNSCC patients, depending on NOTCH pathway mutation status—data extracted from TCGA database. *NOTCH1-4* mutations are not shown in this figure but serve as the basis for comparisons. (**A**) Most recurrent mutated genes in HNSCC show similar mutation frequencies as NOTCH-pathway related genes (compare). Some gene mutations, however, including in caspase 8 (*CASP8*), LDL Receptor Related Protein 1B (*LRP1B*), and FAT Atypical Cadherin 4 (*FAT4*), show significant enrichment (indicated by *) in tumours that harbour Notch pathway mutations (red bars), versus those that do not (blue). This may indicate functional synergism of mutations in these putative tumour suppressors with oncogenic changes to Notch pathway activity. (**B**) A spectrum of additional somatic gene mutations was found significantly enriched (*) in HNSCC tumours that show mutations in NOTCH receptors and ligands, including established Notch-related driver genes such as *FBXW7*, *AJUBA*, and *EP300*. This includes mutations in putative tumour suppressors *KMT2A* and *KMT2C* (Lysine Methyltransferase 2A and 2C), which were previously associated with Notch pathway regulation in other cancers.

All SCCS, but in particular ESCCs and LSCCs show patterns of genetic alterations that are strikingly similar to HSNCC [4], including frequent mutations in the Notch receptors 1–3 [5]. This likely results from intrinsic similarities in the tissues of origin, in particular differentiation-defining DNA methylation marks, which are retained in oncogenic progression and continue to maintain lineage-specific tissue maturation [6]. These analyses also confirmed that “squamousness” is a common and unifying differentiation program that related tumour types, and the normal tissues they originate from, share throughout the most advanced stages of cancer progression [7]. “Squamousness” does not only include similar morphologies in both normal and transformed tissues, but also similar patterns of genetic and epigenetic alterations. In HNSCC, the spectrum includes a very characteristic set of recurrent mutated genes. The COSMIC database of somatic gene mutations in cancers currently (https://cancer.sanger.ac.uk/ (accessed on 17 November 2021)) includes a total of 5888 upper aerodigestive tract cancers, of which 4713 are classified as HNSCC. The most common mutations are in *TP53* (42%), *PIK3CA* (10%), *NOTCH1* (17%), *CDKN2A* (10%), *FAT1* (17%), *LRP1B* (15%), *KMT2D* (13%), *HRAS* (6%), *CASP8* (9%), *EGFR* (4%), *KMT2C* (11%), *NOTCH2* (9%), *FBXW7* (6%), *NSD1* (8%), *FAT4* (9%), *PTEN* (4%), *EP300* (6%), *FGFR3* (4%), *MET* (4%), and *ARID2* (5%). Analysis of large-scale genome-wide expression and mutation databases, such as the TCGA [8,9], show similar mutation frequencies for different HNSCC cohorts. However, the characteristic mutation spectrum may differ, for example, depending on the presence or absence of mutations in Notch-related genes (Figure 1) in the TCGA database [10,11]. This includes, apart from the Notch receptors 1–4, and the five Notch ligands also the genes *AJUBA*, *FBXW7*, and *EP300*, for which lower, but still significant mutation frequencies are observed in HNSCC. Figure 1A shows how absence or presence of Notch-related mutations—found in approximately 49% of the tumours—affects the frequency of mutations in other, probably not Notch-associated genes. The Notch receptors themselves do not appear in this ranked analysis. Most mutation frequencies are not significantly different between these groups, except for *LRP1B*, *CASP8*, and *FAT4* (indicated by *). This may suggest that other, frequently mutated genes such as *LRP1B* or *CASP8* may have unknown functional connections to Notch signalling. In fact, the functionally similar low-density-lipoprotein-receptor-related protein 1 (*LRP1*) gene [12] has recently been described as a mediator of Notch signalling inhibition. LRP1 was shown to decrease tumourigenesis in leukaemia models; pointing to the possibility that such a functional connection to Notch may also exist for *LRP1B*. Figure 1B illustrates other, highly significant differences in mutation frequencies between the Notch-mutated and the non-mutated subgroups. In particular, epigenetic modifiers such as *KMT2A*, *KMT2C* and *JMJD1C* are more frequently mutated in cancers that harbour mutations in known Notch-associated genes such as the four Notch receptors themselves. Most of these differences are statistically significant (again marked by *), pointing towards putative functional, but not yet explored molecular connections of these genes with aberrant Notch functions.

Another common feature, shared by squamous cell carcinoma of the cervix (but not oesophagus and lung), is the presence of human papillomaviruses (HPV). HPV has now been identified as a central risk factor for the development of HNSCC, together with excessive alcohol drinking and cigarette smoking. It is apparent that HPV positive (HPV^+^) HNSCC, predominantly in the oropharynx, behave significantly different from HPV negative (HPV^−^) counterparts; not only in terms of survival and response to therapies [13,14,15], but also concerning genetic [16], epigenetic [14,17,18], or protein expression profiles [17]. Additionally, therapy recommendations are different for patients with HPV^-^ in contrast to patients with HPV^+^ tumours. For example, HPV^+^ tumours tend to respond better to radiotherapy [18,19], which significantly reduces the risk of regional recurrence. Simultaneously, it appears that radical neck dissection as therapy of choice for recurrent metastatic HNSCC is not recommended for HPV^+^ tumours, but appears unnecessary [20]. HNSCC patients with HPV^+^ tumours show better overall survival (OS) and disease-free survival (DFS) in clinical oropharyngeal squamous cell carcinoma (OPSCC). HPV status is one of the most relevant predictive factors for patient stratification and individual risk assessment [20,21]. There are numerous studies, investigating the molecular and transcriptional differences between HPV^+^ and HPV^-^ tumours [20,22], which have identified potential molecular mechanisms affected by HPV status. This includes marked differences in the tumour immune environment, between HPV^+^ and HPV^-^ tumours, since HPV^+^ tumours typically show an inflamed tumour microenvironment (TME) [23]. A number of studies have evaluated differences in the number of immune cells in HPV^+^ versus HPV^-^ tumour tissues, including tumour-infiltrating lymphocytes (TIL) [24,25] and B-cells [22], or the differential expression of immune checkpoint receptors PD-1 and PD-L1 in these tumours [26,27], often summarized as the tumour immune microenvironment (TIME). Luo X-J et al. identified distinct prognostic miRNA signatures of HNSCC with or without HPV infection, with potentially prognostic and diagnostic significance. They showed that immune-related pathways in HPV^+^ tumours were mainly associated with low-risk scores. HPV^−^ tumours, with characteristic high-risk scores, showed instead upregulation of metabolic and other vital oncogenic pathways [28]. It has further been shown that targeting the HPV16/18 viral oncogenes or neoantigens E6 and E7 by immunotherapy in HPV^+^ tumours generated durable immune responses in the tumours, adding these proteins to the list of most promising targets in HNSCC.

High risk (HR) HPV found in head and neck cancers include mainly the types HPV16, 18, 31, 33, 35, 39, 45, 51, 52, 56, 58, 59, and 68. HPV18 is the most common genotype found in HNSCC, with a prevalence of 14–21.7%. Furthermore, HPV 33 and HPV 35 both account for approx. 4.5%. The most frequently mutated genes in HPV^-^ OPSCC are *TP53*, *CDKN2A*, *NOTCH1*, *FAT1*, and *PIK3CA*, followed by Lysine Methyltransferase 2D (*KMT2D*; see also Figure 1). Most of these genes have a direct relation with receptor tyrosine kinases (RTK) downstream signalling, most prominently the EGFR and its downstream effectors RAS/PI3Kinase/AKT/mTOR pathway, and thus activation of cell cycle progression and proliferation. In this function, links to Notch signalling may become most relevant, as we will outline in later sections, e.g., related to EGFR and PI3Kinase pathways. Similarly, mutations in *let-7* and *FGFR3* can be frequently observed in HPV^-^ HNSCC, again linked to the RTK/RAS/PI3K and the Notch signalling pathways.

### 1.2. Introducing the Notch Signalling Pathway in HNSCC

One of the central pathways most frequently affected by DNA-copy number variations and point or splicing mutations in HNSCC is Notch signalling. Mutations in the four receptors *NOTCH1-4* are found frequently in all SCCs, most commonly in *NOTCH1* (17%) and *NOTCH2* (in 9% of HNSCC cases in the COSMIC database, see Figure 1). The Notch pathway is an evolutionarily highly conserved cell signalling sequence, and present in almost all multicellular animals, from hydra to mammals [29]. Mammals possess four different Notch receptors, *NOTCH1-*4, all of which have been identified as mutated—with very different frequencies—in diverse human cancers [30]. Notch proteins are a family of type-1 transmembrane proteins with large extracellular and intracellular domains, which form the core components of the Notch signalling pathway. Simultaneously, there are five Notch ligands, which can bind to any of the four Notch receptors. Ligands are also expressed in most human tissues, including cancer tissues [31]: these are the Jagged 1 and 2 proteins (JAG1 and JAG2), and the delta-like ligands (DLL1, DLL3, and DLL4). Notch ligands are only very rarely mutated, deleted or amplified; but can be over-expressed in some cancers, including HNSCC [32,33]. For example, overexpression of the ligands JAG1 and JAG2 is frequently found >30% of HNSCC, followed by activation and overexpression of the downstream Notch downstream-effectors *Hes1* and *Hey1* [34]. Yet, the Notch ligands have only recently become interesting as potential targets in cancer therapy [35]. Like their receptors, also the DLL/JAG ligands are highly conserved in all mammalian species. Effective signalling of Notch receptors is normally triggered via direct cell–cell contacts, in which the transmembrane proteins of the signal-sending cell (e.g., an endothelial cell expressing Jagged1), form direct or juxtracrine contacts to one of the four Notch receptors at the signal-receiving cells [36]. This basic, mechano-sensing concept is also conserved from insects to mammals. Triggering the downstream signal then induces proteolytic cleavage of the membrane-bound Notch receptors, directly at the cell surface. This is achieved by a series of proteolytic events involving either gamma-secretase (γ-secretase), and/or ADAM10 or ADAM17 metalloproteases, which are sometimes mutated in HNSCC [37]. The γ-secretase is a high molecular weight complex minimally composed of presenilin (PSEN1 and 2), Nicastrin (NCSTN), anterior pharynx defective 1 (APH-1), and Presenilin enhancer 2 (PEN-2). Upon cleavage, the NOTCH intracellular domain (NICD) is released and translocates to the nucleus, where it binds to the CSL/RBPJ transcriptional regulator complex and modifies gene expression in a complex fashion [38]. This further includes transcriptional activators and repressors [39], including epigenetic modifiers such as the histone acetylase EP300 [40]. EP300 is also mutated at comparably high frequency in HNSCC and ESCC (up to 17%; [4,41]), which validates the functional relevance of these epigenetic interactions as downstream component of Notch signalling and transcriptional regulation in HNSCC tumour initiation and progression [40]. In the absence of the NICD, the RBPJ complex typically represses promoter activity via the complexes formed with transcriptional co-activators and repressors, and additionally though recruitment of a histone deacetylase-containing complex. Upon nuclear import and binding to the NICD, the NICD/RBPJ *kappa* complex overcomes this repression and activates the transcription of a signature of characteristic Notch target genes. These have been comparably well understood and are considered prognostic (bio-)markers for Notch activity [42,43]. The “usual suspects” within this Notch signature include genes such as *Hey1* and *Hes1*, *Myc* or Cyclin D1 (*CCND1*). Apart of the signalling molecules themselves, many of the functional consequences of recurrent NOTCH1-4 receptor, Notch-associated gene mutations, or altered Notch pathway activities in the context of living cancer tissues remain poorly understood. This does not only apply in HNSCC, but specifically in relation to therapy responses or failures. For many of the possible roles of the Notch signalling pathway assigned to cancer initiation and development, truly convincing and validated functions are still missing; this also occurs in HNSCC. The Notch signalling pathway plays a complex, pleiotropic and bimodal role in HNSCC: NOTCH receptors and/or the Notch signalling cascades can act as both tumour suppressor and proto-oncogene/tumour promoter—strongly depending on the composition of the TME and ECM, tumour size, stage of tumour progression, tumour grade, and the genetic wiring of these tumours. The notion that Notch signalling may also be activated in an oncogenic fashion is relatively new to the field of HNSCC, but is reality in haematopoietic tumours such as T-ALL. Any context-dependent activation of Notch signalling in HNSCC is more difficult to functionally validate compared to loss-of-function, often truncating mutations, and likely involves a complex network of biochemical and genetic interactions within the tumour tissue. Some of these potential targets are invisible and hidden from our eyes, we may only be able to detect significant phenotypic changes that relate to increased tumour cell plasticity and changes in cell differentiation, or determination. Thus, it is still unclear how to effectively target the Notch signalling pathway in cancers: Should we aim only for the ligands, or the receptors? Maybe, individual receptors represent potent targets, but only after detailed functional analyses of the Notch activities in a patient’s tumour tissue? Currently, targeting Notch signalling is largely shooting in the dark, since we poorly understand the physiological context.

### 1.3. The Tumour Microenvironment (TME) and Intra-Tumour Heterogeneity (ITH) as Moving Targets: Notch, Tumour Heterogeneity and Cellular Dynamics

Striking differences in tissue architecture, morphology and composition can be observed not only between different patients with HNSCC, but also within larger biopsies from the same patient. Differences between metastatic lesions of the same patient can be particularly pronounced, as a result of what is now understood as increased tumour evolution [44,45], a driving factor of progression, therapy failure and metastasis. Recent findings have highlighted the high physiological relevance of intra-tumour heterogeneity (ITH) as a central, defining characteristic also in HNSCC [46,47]. Marked changes in tumour heterogeneity are a prominent feature observed between primary tumours and lymph node or distant metastasis [48], also between different metastases of the same tumour [49]. Apart from HPV^+^ or HPV^−^ tumours, HNSCC can show remarkable differences in histology, mainly due to various degrees of epithelial (de-)differentiation or maturation of the cancer cells [50], derived from squamous epithelial cells or keratinocytes. These pronounced and characteristic differences in histopathology have been successfully utilized for **tumour grading** and prognosis. Other morphologic phenotypes that are systematically evaluated for histopathological grading include, for example, stromal features, used for **stromal categorization** [51]. This may rely on distinguishing different degrees of **desmoplastic reaction** in HNSCC tumour tissues, and classification of mature and immature, keloid-like collagen and myxoid stroma features [52,53,54]. The stromal compartment, in addition to the tumour cells, may also be interesting as a target for chemotherapies [55,56], specifically tumour- and stromal-cell plasticity [55]. Many of the histopathologic features currently associated with tumour cell plasticity, and processes such as EMT are only found in some regions of a tumour biopsy, but entirely missing in others. This specifically applies to the **invasive front** of tumours. Here, we enter the territory of Notch signalling (and Notch-related mutations) in HNSCC tumour progression. Some of the most intensely investigated Notch-related phenotypes observed in cancers, including HNSCC, are very closely related to the TME, and the generation of ITH. Increased tumour cell plasticity thus contributes significantly to (a) tumour initiation and progression, (b) acquired or secondary resistance to therapy, (c) formation of de-differentiated cancer stem cells (CSC, or “stemness” of cancer cells), or (d) promoting mesenchymal trans-differentiation of transformed epithelial cells. Notch signalling has also been recently identified as (e) critical for defining the perivascular stem cell niche in breast and other epithelial cancers [57,58]. However, comparable studies in HNSCC are currently missing. Notch plays decisive roles, although not always completely understood, in almost all of these processes, which we will discuss in detail throughout this article. The most relevant, Notch-associated phenotypic and/or genetic processes include: (1) angiogenesis, (2) generating enhanced tumour cell plasticity, (3) promoting the invasive properties of cancer cells, and (4) defining stem cell biology. This is completed by (5) provoking changes in tumour cell metabolism and (6) regulating the cell cycle, which is critical for HNSCC chemotherapy.

## 2. Targeting Oncogenic Pathways in HNSCC

### 2.1. Current Status of HNSCC Therapy

In comparison to other neoplasms, such as breast cancers, only very few new drugs have entered clinical practice of advanced HNSCC patients in the past 10–20 years; radiation therapy remains an important pillar. Approximately 40–50% of these therapies fail, resulting in recurrence and/or metastasis or tumour relapse. Most of these advanced patients eventually die from disease-related complications. The current standard for HNSCC treatment primarily relies on surgery and radiation. Only for advanced HNSCC patients, definitive chemotherapy is considered, which often includes palliative therapies with no intention to cure. These progressive regimens are essentially based on “traditional” anticancer drugs for adjuvant or neoadjuvant chemotherapies such as cisplatin/carboplatin (CDDP), 5-fluoro-uracil (5-FU), and Taxanes such as Paclitaxel and Docetaxel. Only in the case of recurrent or metastatic HNSCC (R/M HNSCC), **Cetuximab**, small molecule EGFR inhibitors, and novel immune checkpoints inhibitors such as **Pembrolizumab** and **Nivolumab** are included in the treatment [59]. However, these targeted therapies are also rarely curative and will be discussed further below. The immune checkpoint inhibitors have recently been approved also for treatment of earlier tumour stages, for example in smaller, resectable lesions [60].

The primary, initial treatment guidelines for HNSCC vary, according to the location of the tumour and stage of the disease, and can be improved with metronomic chemotherapy [61]. The mechanism of action for all “classic” chemotherapeutics is typically based on cytotoxicity, targeting generic, non-cancer specific processes such as mitosis and DNA damage/damage response. These broad mechanisms primarily focus on reducing cancer cell growth and proliferation or inducing programmed cell death/apoptosis, with a very narrow selective margin between neoplastic and normal cells. This almost blind-folded “carpet bombing” strategy results in severe adverse events [62], which are particularly incapacitating for patients treated with combined chemoradiotherapies. The result is a significant loss of quality of life (QoL) for the patients. It can also be extremely frustrating for clinicians, not being able to use more targeted, specific, and well-tolerated drugs, or synergistic drug combinations. It remains difficult to select more promising therapies over others, based on reliable evidence-based patient stratification. In HNSCC, it is particularly difficult to make informed therapy decisions, based on any solid evidence (=predictive panels of biomarkers, tumour histology, staging and grading, liquid biopsies, etc). Despite the limited, but undoubted survival benefits that some patients may derive from combination chemotherapy, there is an urgent need to promote (a) the development of better, more specific and targeted anti-cancer agents for HNSCC patients; (b) to develop predictive methods for personalized therapy planning and patient stratification; and (c) to improve the efficacy of the few targeted drugs, in particular Cetuximab and the immune checkpoint inhibitors (ICI).

### 2.2. Tools for Personalized Medicine in HNSCC and Notch Signalling

Currently, decision-making, and patient stratification for various forms of therapy by clinicians are still very much limited. There are only very few, incompletely evaluated tools available to stratify HNSCC patients, for example according to genetic parameters. We will see that the Notch signalling pathway may play a very critical and significant role for the purpose of personalized medicine: no other pathway is as intricately connected to “phenotypic” differences in the architecture or histology of tumour tissues. Probably no other pathway is as closely related to increased tumour cell plasticity and its effects on chemo-and/or radiosensitivity, development of secondary (acquired) drug resistance, invasive properties of tumour cells, neo-angiogenesis and vascular mimicry, relapse, dormancy and tumour stem cells. These mechanisms essentially define the challenges clinicians face considering personalized medicine: how can we possibly predict the response of a patient’s tumour cells to various therapies, when these cells show such a high capacity to evade cytotoxicity and induced cell death? Tumour cell survival, or therapy evasion may to a large part be conveyed by (hyper-)activation of signalling pathways that have direct control over cell fate and commitment, differentiation versus proliferation, and stem cell properties. No validated tool for personalized medicine has been approved for clinical use, to identify beneficial, molecular, or targeted versus standard-of care therapies or potentially beneficial drug combinations to be prioritized over others. Nevertheless, different molecular subtypes have recently been identified in HNSCC [46,63,64] and nasopharyngeal carcinomas [65], mainly based on histological evaluation, single-cell sequencing, and genomic analysis. One critical distinction has been mentioned: between HPV^+^ and HPV^−^ cancers. HPV^+^ cancers characteristically show a better response to therapies, are less aggressive, and show better outcomes [66].

Apart from predicting chemosensitivity, it is probably even more clinically relevant to assess the likelihood that a tumour responds to radiotherapy, the most common first-line therapy in HNSCC. The molecular and cellular basis of radiosensitivity versus radio-resistance has been intensely investigated in HNSCC since the 1990s. For example, radiosensitivity has been correlated with numerous clinical and morphological/histological factors, including microvessel density [67], hypoxia [68], expression of mesenchymal or stromal biomarkers such as Fibronectin [69], the presence and proliferation of cancer-associated fibroblasts (CAFs) [70], and key signalling pathways such as EGFR, mTOR, and PI3Kinase [71]. If and how Notch signalling may play a role in these processes has only more recently entered clinical research.

In HNSCC, the staging and grading of cancer tissues based on histopathology has a limited, but growing value for estimating the potential of a tumour to progress to advanced disease [52,54]. Improved histopathologic grading begins to play a major role in clinical decision making, and may become more important—provided major technical shortcomings regarding their routine use for prognostic patient stratification, and issues with the standardization and reproducibility of the methods can be resolved [72]. Most relevant in this context is the observation that many of the phenotypic changes observed in more aggressive cancers, such as high tumour grades, may be directly or indirectly related to the activity of the Notch signalling pathway, and related mechanisms such as Wnt signalling. Improved, image-analysis based quantification systems in specific histopathologic grading methods may significantly enhance the predictive potential of advanced histopathology assays [73], based on these phenotypic changes. The use of artificial intelligence (AI) to these tasks is generally on the rise, as reviewed in [74]. In particular, the phenotypic features mentioned may be addressed by advanced, improved and more robust AI-based methods in the future, combining heuristics, supervised and unsupervised learning methods. Similarly, deep learning-based methods may play a significant role for the same purposes in the future.

For example, the depth of tumour cell invasion can be assessed by quantifiable morphological criteria, such as “worst pattern of invasion”, “tumour budding” [75,76], and “cell nest size” [75]. These are functionally related, defined histopathologic features with a particularly high potential for improved and prognostic patient stratification. These morphologic features likely relate to the enhanced invasive or motile potential of “budding” tumour cells within the tumour tissues. They are likely defined by few central processes (including Notch signalling) that promote tumour cell plasticity, including de- or trans-differentiation processes, such as epithelial-to-mesenchymal transition (EMT). Plasticity-related morphologic features such as “tumour budding” are outstanding parameters of inter-tumour heterogeneity, and have the potential to predict at least locoregional recurrences in oral cancers [52].

### 2.3. Novel Therapeutic Agents—Overview 

Few novel forms of therapy (apart from immune checkpoint inhibitors) have entered the field of HNSCC therapy during the past decades. Naturally, there is vivid early stage drug discovery and translational research ongoing on, some of which is summarized here. This is combined with intensive clinical research on the use and benefits specifically of various targeted therapies, and drug combinations. For example, with the success of the first immune checkpoint inhibitors, there is now renewed interest in developing and testing recombinant, humanized, monoclonal antibodies—such as **Cetuximab**, **Panitumumab**, **Pembrolizumab**, **Nivolumab**, **Sintilimab**, **Durvalumab**, **Ipilimumab**, **Tremelimumab**, **Cixutumumab**, **Mogamulizumab**, **Utomilumab**, and **Rituximab—**covering a broad spectrum of molecular targets (summarized in Table 1)**.** Simultaneously, there has been continuous interest in the development and clinical testing of target-specific, low molecular weight inhibitors such as **Vorinostat**, **Romidepsin**, **Sodium dichloroacetate**, **Rapamycin**, **Methotrexate**, **Vinca alkaloids**, **Axitinib**, **Palbociclib**, **Acalabrutinib**, **Alisertib**, **Lapatinib ditosylate**, **Dacomitinib**, **Erlotinib**, **Olaparib**, **litronesib**, **Ispinesib**, and **Motolimod** (summarized in Table 1). In addition, some more “exotic” concepts have been developed, including therapeutic peptides such as **Dalantercept**, non-coding RNA (ncRNA) such as **EMD1201081**, and even engineered viruses such as **Talimogene laherparepvec** and **MEDI0457** (known as INO-3112). These are currently in clinical trials to evaluate their use in HNSCC therapy (for more details, compare Table 1).

It is assumed that blocking key oncogenic signalling pathways slows tumour growth by reducing cell proliferation, migration, and angiogenic sprouting, leading to tumour regression or at least reduced aggressive behaviour. Some of these molecular targets induce cancer cell death or activate the host’s immune system to support anti-cancer therapy. Among these novel concepts, there are some developmental drugs in early stages of development, that directly target Notch signalling (although none are clinically tested in HNSCC yet). In addition, some of the other targeted therapies introduced in this chapter, and their respective signalling pathways, have close connections to Notch signalling, without directly targeting the Notch receptors or ligands; but extensive crosstalk between pathways is likely to include Notch signalling. For other drugs and drug targets, connections to Notch signalling may yet be discovered. It is also possible that differential Notch pathway activities in the tumour cells or the TME may indirectly affect the response of tumour cells to these novel therapies; but this field of research is only emerging. Furthermore, combinatorial therapies are conceivable to specifically exploit some of these potentially synergistic mechanisms of action. For example, it is well established that EGFR and Notch signalling pathways are interconnected, and therefore represent dual targets for single or combinatorial therapeutic intervention. Based on scientific evidence from the literature, we present some of the most relevant targets and pathways for therapeutic intervention, with a strong focus on how Notch signalling may be involved as a modifier, modulator, enhancer or to provide physiological context for drug responses. We will also focus on the role that the TME, intra-tumour heterogeneity, and enhanced tumour cell plasticity may have in specific drug responses and the development of resistance; also closely linked to Notch signalling.

### 2.4. Homing in: NOTCH & NOTCH Signalling as a Target in HNSCC

#### 2.4.1. First Act: Oncogenic NOTCH Mutations Found in Diverse Cancers

**Figure 2:**
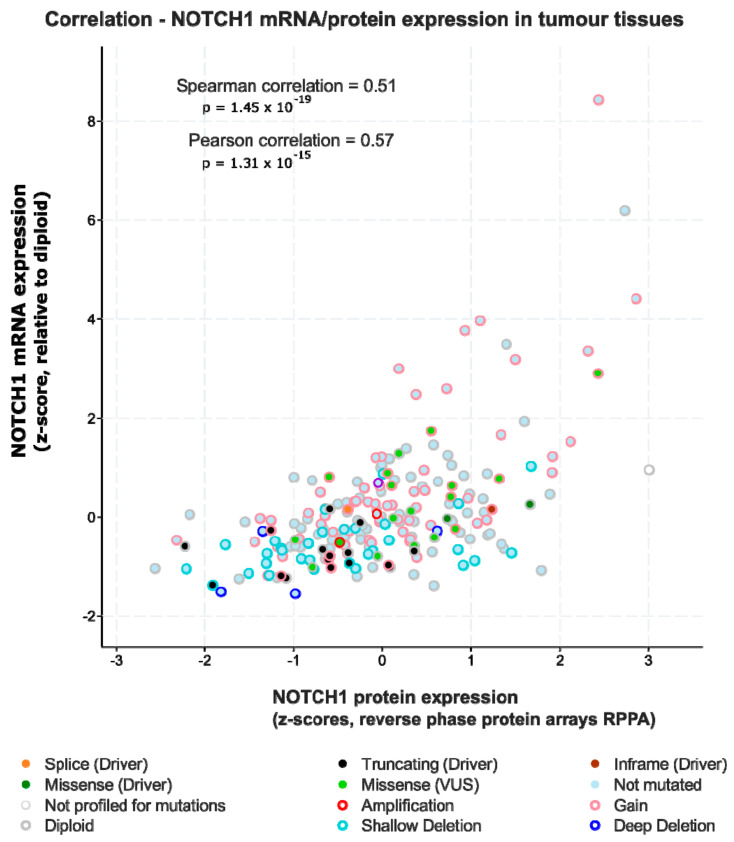
Correlation of mRNA and protein expression of NOTCH1 in HNSCC illustrates the likely bimodal functional activities of this gene, which can act as both tumour suppressor and proto-oncogene. The graph shows the levels of *NOTCH1* mRNA (RNA seq, counts per million reads), plotted against NOTCH1 protein (reverse-phase protein arrays, RPPA; from TGCA “Firehose Legacy” data set). Tumours with NOTCH1 deletion (light and dark blue circles) and/or truncating, loss-of-function mutations (black dots) typically express low to very low ratios of both NOTCH1 mRNA and protein. In contrast, a fraction of tumours (marked by light red circles) show marked over-expression of NOTCH1 mRNA and protein. Missense mutations are rare in these tumours, and no truncating mutations were found.

Aberrations of the four NOTCH receptors have first been identified as initiating and driving mechanisms for the development of haematopoietic malignancies, such as T-cell acute lymphoblastic leukaemia (T-ALL). Since this seminal discovery in 2004 [77], mutations, deletions and amplifications (indels) of NOTCH receptors, mainly of NOTCH1, have been identified in most or all epithelial cancers, including breast adenocarcinoma and various squamous cell carcinomas [30,78]. Mutations in NOTCH1, followed by NOTCH2 and a smaller number of NOTCH3 mutation, are also among the most frequently mutated genes in HNSCC (see also Figure 1). In contrast, NOTCH4 is very rarely mutated. The spectrum of mutations observed in HNSCC further includes additional, putative oncogenic driver mutations in players that may modulate Notch signalling, such as AJUBA, EP300, FBXW7, etc. [79,80]. Early on, *NOTCH* mutation status (regardless of direction of change) was correlated with poor survival of the patients [43,81] (Figure 1 and Figure 2). Interestingly, HNSCC lesions that harbour mutations in any of the central players (*NOTCH1-4*, Notch ligands, and modulators *AJUBA*, *EP300*, and *FBXW7*) show different mutation spectra, for example are enriched in mutations of caspase 8 (*CASP8*) and *FAT4* (Figure 1). This indicates a different genetic (and epigenetic) environment that may be promoted by different DNA alterations, compared to tumours that show absence of Notch-related mutations. The role of epigenetic alterations in initiation and progression of HNSCC has been recently summarized in a detailed review article [82]. To which degree Notch signalling and epigenetic alterations are connected, can only be assumed by the fact that a number of epigenetic modifiers, such as the histone acetylase EP300 [40] which is frequently mutated in HNSCC. Similar connections emerge between Notch signalling activities and mutations in histone methylation-related genes such as the lysine methyltransferase 2, also known as *MLL-1*, *KMT2A/MLL-1*, and other members of the KMT gene family, such as *KMT2C* and *D*, are frequently mutated in HNSCC and show an interesting positive correlation with the presence of Notch mutations (Figure 1B). It has been shown that inhibitors blocking the histone-modulating functions of KMT2A, and related genes potently inhibit tumour cell proliferation in glioma cells, a partly by DNA methylation of *NOTCH1* and *NOTCH3* genomic locus [83]. No comparable data exist from HNSCC tissues or cell lines, but this line of research appears particularly promising. Other research in colorectal cancer has shown similar connections between Notch activity, and KMT2A/MLL-1. High activity of this protein induces tumour cell proliferation and induces a regenerative cell state, with high cell renewal potential that directly correlate with trimethylation of histone 3 at lysine 4 (H3K4me3) [84]. It is likely that mutation, activation, and/or overexpression of KMT2A and potentially some of its relatives KMT2B-D may regulate tumour cell growth and proliferation, stemness and self-renewal potential; this may be linked to increased tumour cell plasticity and stemness. Comparable studies on HNSCC tumour cells, or patient-derived organoids, are currently not available, but may represent an interesting research topic with direct clinical relevance [84].

Early reports concerning the putative function and clinical consequences of Notch mutations (usually NOTCH1) in HNSCC also suggested a possible gain-of-function, or proto-oncogene role, such as the gain-of-functions of mutations observed earlier in T-ALL. Gain-of-function mutations in any of the four NOTCH receptors may be observed in Western HNSCC patients but are probably very rare. In contrast, tumour-promoting NOTCH1 mutations have been frequently identified in the Chinese population [43], for example in oesophageal squamous cell carcinoma [2]. Some of these oncogenic missense mutations have also been functionally analysed. The best understood gain-of-function mutation in NOTCH1 is the recurrent C1133Y point mutation. This specific hotspot has been termed “Abruptex” mutation [85,86] and was shown to promote cell proliferation, invasion, and to induce EMT in OSCC cell lines. The C1133Y mutation leads to failure of protein processing and surface translocation [85], similar to some of the characteristic hotspot mutations found in the PEST domain of NOTCH1 in T-ALL. Functionally, both types of mutations—PEST-domain or Abruptex—result in a constitutively active NOTCH1 protein with an extended nuclear half-life. This protein shows a high potential to activate the EGFR-PI3K/AKT signalling pathway (which, once again, points to the links between EGFR and Notch signalling). The NOTCH1-abruptex mutation further collaborates with the F-box protein FBWX7, a regulator of proteasomal protein degradation, which promotes and potentiates the activity of this NOTCH mutant [86]. The specific selection of otherwise rare, gain-of-function NOTCH1 mutations in Chinese HNSCC patients may be related to different tumour aetiology, as well as specific genetic and environmental factors in these populations. However, more detailed, recent studies based on next-generation sequencing and genome-wide mutation analyses have increasingly pointed to the predominant nature of loss-of-function mutations in HNSCC [87,88,89,90]. It was further reported that high expression levels of *NOTCH1* mRNA in the tumour tissues correlate with improved patient outcomes and longer survival [91], which seemed to corroborate a positive, tumour-suppressor-like function of the Notch pathway, which is then lost by alterations. This potential “**bimodal function**” of Notch signalling specifically in squamous cancers, and HNSCC, was first postulated in 2015 [92]. It now appears that Notch signalling in tumour biology is not as straightforward as it originally seemed, and that NOTCH mutations, and generally aberrations in the Notch signalling pathway, show in fact a very complex and pleiotropic pattern of oncogenic activities. In addition, over-expression of Notch proteins and modifying regulators may play a dominant role in late-stage cancer progression. If and how increased Notch signalling activities—often in the presence of inactivating NOTCH1 mutations.

#### 2.4.2. Second Act: Loss-of-Function Mutations, but Yet, Gain-Of-Function Activities?

The emerging understanding of Notch signalling in HNSCC has been expertly summarized in a series of recent review articles, including [92,93,94,95,96]. The observation of increased Notch activities in advanced, R/M HNSCC, contrasting the frequent loss-of-functions observed in early stage tumours, is perfectly in line with some of the common “areas of expertise” in which Notch excels: A significant part of Notch activities directly relate to controlling cell fate decisions, in particular promoting lineage-specific differentiation of mature tissues and cell functions [97,98,99]. This is especially prominent in squamous tissues/cornified mucosae and may help define “squamousness”. Concomitant loss of terminal (or functional) tissue-specific differentiation is often coupled to hyperproliferation. This is immediately to the benefit of early stage tumour cells that may be arising in otherwise still normal, functional tissues, but struggle to compete with normal, proliferating cells. In this fashion, loss of Notch may provide emerging premalignant, and transformed, early stage cancer cells with a local, selective growth advantage—initiating tumour evolution. Loss of Notch activities may further help overcoming intrinsic cellular control mechanisms such as senescence and contact inhibition [100,101]. This may be simultaneously promoted by other mutational events, such as loss of p53 [102]. In line with this, *NOTCH1* has been identified as a p53-responsive gene. Frequent TP53 mutations, already found in early stage premalignant lesions of HNSCC [103,104,105], would in this fashion result in at least a partial loss of NOTCH1 functionality in these tissues, even without *NOTCH* mutations. On the other hand, Notch functions simultaneously promote stemness of normal and cancer cells, and tumour cell plasticity, EMT, and other, trans-differentiation processes (see Section 3 on tumour cell plasticity and Notch). Additionally, Notch signalling is also critical in controlling blood vessel sprouting and angiogenesis (see Section 4), thus generally supporting later stages of cancer development [93,106,107].

**Figure 3:**
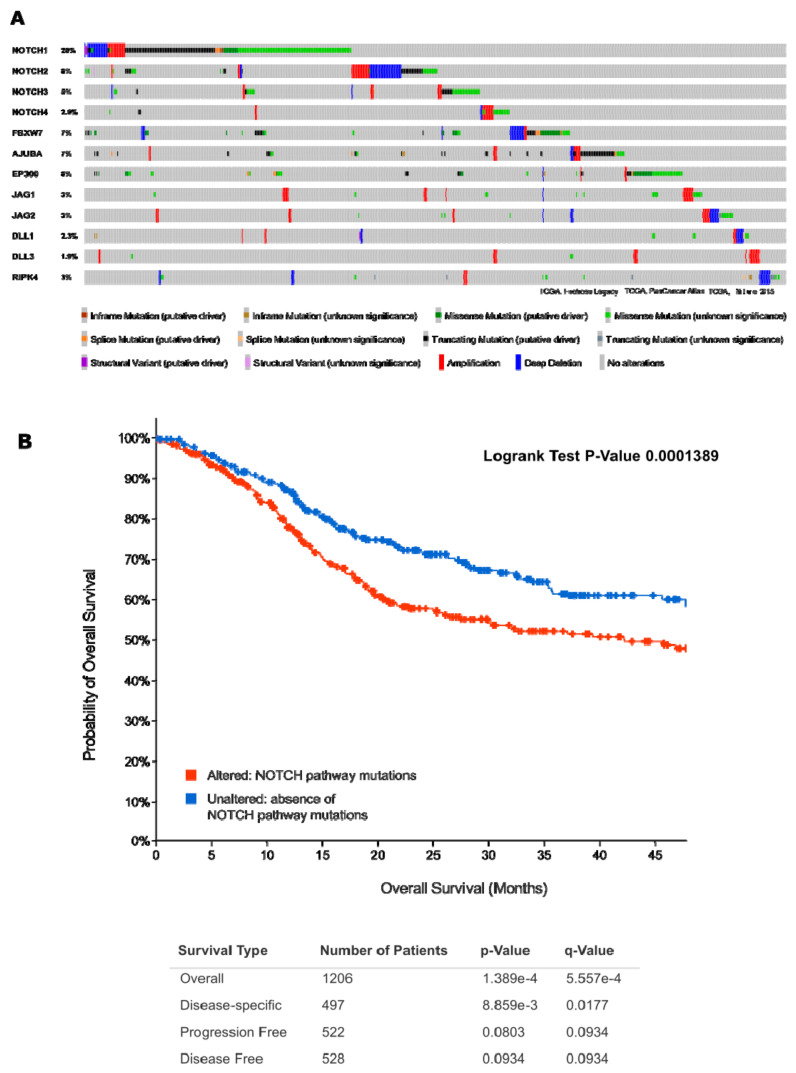
Impact of mutated Notch pathway genes on the survival of head and neck squamous cell carcinoma (HNSCC) patients. (**A**) DNA copy number changes, structural and point mutations of 12 genes central to Notch pathway activity, across 1332 patient with HNSCC from The Cancer Genome Anatomy (TCGA) repository. (**B**) Kaplan–Meier plot indicating significantly different overall and disease-specific survival of HNSCC patients that harbour at least one Notch-pathway related mutation or copy-number change (red), compared to patients without mutations in any of these 12 genes (blue). The same 12 genes were also included in the clinical association studies shown in Figure 4. Conclusion: differential activities of the Notch signalling pathway are likely to affect patients’ therapy response and survival (up to 48 months).

However: A growing number of studies indicate that increased Notch activity may indeed be largely oncogenic in advanced HNSCC [34,108,109]. It is now considered that generalized activation of downstream Notch signalling may promote late-stage cancer progression, regardless of frequent *NOTCH1* mutations, which show an overwhelming negative functional impact. In addition, HNSCC cancer cases have been identified that clearly show amplification of the *NOTCH1* locus on chromosome 9 (see Figure 1), in conjunction with over-expression of *NOTCH1* mRNA and protein (Figure 2). Similar findings exist for *NOTCH2* [109]. Notch protein expression essentially correlates with mRNA expression, as shown by reverse-phase protein arrays (Figure 2). Additionally, the DNA copy number of the NOTCH1 locus, such as diploid versus deep or shallow deletion, and amplification, naturally correlates to some degree with Notch1 mRNA expression. In HNSCC cases with high *NOTCH1* mRNA/protein expression, no missense or nonsense mutations are found, especially no truncating and damaging nucleotide exchanges. In contrast, the existence of cases that show amplification and overexpression of *NOTCH1* or *2* is supported by genome-wide expression and copy number variation studies (TCGA, The Cancer Genome Atlas; Figure 3). To further validate this, in several functional studies, *NOTCH1* signalling has been shown to favour the development and progression of HNSCC [88,110]. As expected, this refers mainly to tumours in which Notch signalling may specifically support the induction and maintenance of the self-renewal of cancer stem cells (CSC), and/or cases with a high degree of hybrid/partial EMT (see below). As if to confirm these assumptions, other studies have shown that increased *NOTCH1* signalling and enhanced stemness can render tumour tissues less sensitive to standard chemotherapeutic agents, such as cisplatin, docetaxel, and 5-FU [111]. Additionally, in oral squamous cell carcinoma (OSCC), *NOTCH1* signalling has also been convincingly shown to promote metastasis, possibly via the support of EMT [112,113]. The impact of the presence or absence of Notch-pathway related mutations on patient disease-free and overall survival is further illustrated in Figure 3; although in this case we cannot clearly distinguish between gain- or loss-of-function mutations. Some of the studies on other NOTCH receptors beside *NOTCH1* are of highly controversial nature, pointing to specific and individual roles of these receptors. However, most data on *NOTCH2-4* are typically of more episodic nature. For example, inhibition of *NOTCH3* signalling was shown to promote EMT [100], and increases cellular resistance to chemotherapy in HNSCC [114]. Like *NOTCH1*, the *NOTCH4* signalling pathway can also be over-activated in HNSCC, resulting in an increase in cellular resistance to cisplatin, accompanied by increased EMT characteristics, higher invasive potential, and acceleration of proliferation [93]. Additionally, the *HEY1* overexpression induced by *NOTCH4* signalling promotes EMT in HNSCC cells [115].

Independently, the frequent phenotypic gain-of-function of Notch signalling in HNSCC is convincingly corroborated by treating established HNSCC cell lines and/or mouse xenografts with Notch inhibitors, such as the potent FLI-06 [80,116]. This effectively blocks cell proliferation and induces cell apoptosis [80]. Simultaneously, expression of *NOTCH1* mRNA and protein, Notch downstream activity, and expression of Notch target genes are all inhibited by FLI-06. One would expect that the same applies for a large fraction of patients’ tumours, when treated with such drugs? There are currently no new, experimental Notch inhibitors in clinical studies for HNSCC, which is the case for other tumour entities. Altogether, no “classic” Notch inhibitors such as GSIs have been approved for any cancer type.

Then, in some studies, increased Notch activity has been associated mainly with alterations observed in the stromal fibroblasts or other cell types [117], often focusing on Notch ligands. Apart from the NOTCH receptors themselves, it is also important to address the expression and function of the five Notch ligands. In HNSCC, some attention has been granted to JAG1 and JAG2, which were found overexpressed in some tumours [43,118]. Similarly, the Notch target genes *Hes1* and *Hey1* were found overexpressed in a significant portion of HNSCC tumours, again suggesting hyperactivity of Notch signalling in these tumour tissues [34,115,119]. Overexpression of this characteristic functional “Notch signature” was found in up to 32% of all HNSCC patients, but not in those carrying inactivating *NOTCH1* mutations. This signature, largely driven be genes such as *Hey1*, *Hes1*, *Myc*, and *cyclin D1*, also correlates with overall and disease-specific patient survival. It remains currently unclear which mechanisms exactly activate Notch signalling in these cases, and precisely which downstream signalling molecules in the Notch pathway may be involved. These data also indicate a likely bimodal pattern of Notch pathway activation in HNSCC, in which inactivating mutations in the receptors themselves only represent one of the receptor subtypes observed.

Not surprisingly, there are numerous connections between HPV^-^mediated molecular mechanisms, and Notch signalling. Recent studies have shown that a number of molecular signalling pathways are differentially affected in HPV^+^ vs. HPV^-^ tumours [120]. For example, EGFR, IL-1, IL-6, JAK-STAT, Wnt, Notch, and ESR1 signalling pathways were all found to be consistently downregulated in HPV^+^ cases, but not in HPV^-^ tumours. The Notch signalling pathway has also been demonstrated to modulate HPV oncogenes in HPV^+^ cancers, or vice versa—there are mutual molecular and cellular relationships. HPV5, HPV8, and the β-HPV types can weaken NOTCH activity, probably by manipulating the NOTCH-associated transcriptional machinery. The E6 oncogene of the β-HPV subtypes, for example directly binds to the Notch-associated factor *MAML1* [121]. This hinders the interaction between MAML and NICD, thus causing the loss of expression of NOTCH target genes such as *HEY1* and *HES1*. Additionally, the oncoproteins of high-risk α- papillomaviruses can impede NOTCH signalling indirectly, for example through degradation of the TP53. In contrast, increased NOTCH1 activity can negatively modulate E6 expression by inhibiting the AP1 transcription factor complex. High-risk HPV oncogenes may also target other p53 family members, such as TA*p63*β; and indirectly modulate NOTCH1 expression and activity in this way [121].

#### 2.4.3. Act 3: What Exactly Drives Activation of Notch Signalling in HNSCC?

The recent identification of a large number of Notch-related rare mutations in HNSCC [37], and specifically the identification of additional Notch-related genes such as *AJUBA*, *RIPK4*, and *ADAM10* may help to clarify these unclear mechanisms in the future. The detailed analysis of the molecular function of Notch-related, mutated and/or overexpressed target genes such as *AJUBA* [79] (part of the gene set shown in Figure 3A) will likely contribute to a broader picture of Notch pathway activity in the cancer cells themselves, but also in adjacent stromal and endothelial cells, and the immune cell infiltrate. The spectrum of genetic alterations (mutations, amplifications, deletions, epigenetic changes, etc.) in HNSCC harbours a number of additional genes that feed into the Notch signalling pathway, and which are mutated at frequencies between 5 and 15% [4,122]. This includes genes such as the tumour-suppressor gene *FBXW7* (mutated in 7–8% of HNSCC), a member of the F-box protein family, which acts as a central part of the E3-ubiquitin ligase complex SCF. The SCF complex ubiquitinates proteins and triggers proteasome degradation of intracellular proteins, including the NICDs of NOTCH1-4 themselves. FBXW7 mutations (Figure 5), therefore, stabilize and promote the transcriptional activities of the NICD, and directly contribute to increased Notch pathway activity [123]—resulting in a net gain of Notch activity. This is further validated by observations that mutations or low expression levels of FBXW7 correlate with poor patient outcome and survival [4,124]. An isoform of FBXW7 has been identified to specifically promote the unique gain-of-function or “Abruptex” mutations found in some Chinese patients with oral cancer [86]. FBXW7 is also involved in the maturation of normal squamous mucosa [125]: it was shown to regulate the decision of keratinocytes to either proliferate or differentiate by the proteasomal degradation of c-Myc and NOTCH proteins. This elegantly confirms the key role of the Notch signalling pathway in tissue formation, differentiation, and homeostasis, especially in healthy squamous mucosa. Furthermore, FBWX7 and the NICD both interact with the histone acetylation protein EP300 and may therefore impact on epigenetic modification of potential Notch target genes [40]; an observation from haematopoietic neoplasms that yet has to be confirmed in HNSCC. Last not least, an isoform of FBXW7 (FBXW7β) has been identified as an effective inhibitor of the rare “Abruptex” gain-of-function mutations in *NOTCH1* [86], by decreasing the half-life of the mutated, hyperactivated NICD within the nucleus. This clearly explains why FBXW7 mutations or deletions promote late-stage HNSCC progression and growth as well as directly argue for a role of NOTCH1-pathway activities in this cancer entity. Instead of targeting NOTCH receptors, the most common strategy, it may be worthwhile considering how one could pharmacologically interfere with, or functionally revert mutations in genes such as *FBXW7* and *AJUBA* in some patients. Similar concepts may, in extension, also apply for other Notch-modulatory genes found mutated in HNSCC, including the epigenetic modifier EP300 [40].

### 2.5. NOTCH and EGFR Signalling

#### 2.5.1. Cetuximab and EGFR Pathway Activity in HNSCC

Epidermal growth factor receptors (EGFRs) and/or EGFR downstream signalling cascades have long been understood as essential for the development of squamous cell carcinomas [126]. Based on experimental data originally from cell lines, HNSCC cells were further shown to highly overexpress EGFR (also ErBB1 or HER). This was later confirmed by immuno-histological and genetic studies, indicating that HNSCC harbour relatively frequent alterations (such as amplifications, rearrangements, and missense mutations) in the *EGFR* gene. However, only approximately 4% of all HNSCC show *EGFR* mutations. Genes downstream of the EGFR pathway are more frequently mutated: The somatic alterations most relevant for EGFR signalling occur in *PIK3CA (10%)*, *KRAS (6%)* and loss-of-function mutations or deletions of *PTEN (4%)*. Furthermore, mutations in *PIK3CA* and *KRAS/HRAS* as well as loss of PTEN protein expression correlate with poor progression-free survival in HNSCC patients [127,128]. In HPV^+^ tumours, functionally similar mutations in KRAS are frequently found, while HRAS mutations are rare [17].

These mutations are typically associated with characteristic protein expression (or protein phosphorylation) patterns that are responsible and at the same time a hallmark of modified EGFR pathway activity. Not surprisingly, genome-wide DNA sequencing showed that the sensitivity of HNSCC patients to cetuximab is not only dependent on EGFR mutation status, but generally fluctuates with mutations in these additional EGFR pathway genes. A number of newer studies have also confirmed that EGF receptor expression and downstream signalling activity are not well correlated [129].

EGFR signalling has become a key pathway for targeted therapies for HNSCC [130]. Presently, and despite some considerable side effects [59], the most advanced and characterized EGFR inhibitor is **Cetuximab**. This recombinant, humanized antibody has been approved by FDA and EMEA and is the most widely used molecularly targeted drug for the treatment of HNSCC. Mechanistically, cetuximab blocks the extracellular domain of EGFR dimers, located at the plasma membrane, inhibits its signalling, and leads to rapid receptor internalization. Additionally, upon binding to the receptor, Cetuximab potently induces antibody-dependent cellular cytotoxicity, resulting in subsequent destruction of the tumour cells [130]. Cetuximab has also been shown to reduce the secretion of pro-angiogenic factors such as VEGF (vascular endothelial growth factor), but its use for therapeutic inhibition of angiogenesis at least in monotherapy is questionable [131]. Unfortunately, monoclonal antibodies such as Cetuximab and small-molecule tyrosine kinase inhibitors (TKI) did not fulfil the high expectations that had been associated with these targeted drugs. Cetuximab initially showed promising results in clinical trials. However, it eventually failed to show a very significant impact in the clinic, mainly due to its poor results in locally advanced HNSCC, despite some rare, good responders [132]. The current limitations of anti-EGFR therapies by Cetuximab or other drugs likely relate to the mutation status and activation of the EGFR pathway in the patients’ tumour cells. It is not surprising that blocking EGFR activity is not entirely effective if the same cells also show constitutive activation of the downstream signalling mechanisms, e.g., via KRAS/HRAS mutations, loss of PTEN, and overexpression of PI3Kinase via PIK3CA mutations. Furthermore, overexpression of EGFR (or HER2/ERRB2) is not characteristic for the majority of tumour samples obtained from HNSCC patients [133], and EGFR downstream activity in tumour tissues is significantly below the levels observed in HNSCC cell lines. Recent findings showed that a specific subgroup of HNSCC patients with alterations in NOTCH, RTK/RAS/MAPK, and TGF-β pathways that had a significantly negative impact on disease-free survival [134], pointing to a link between these signalling pathways in tumours (as discussed below).

Nevertheless, the primary use of Cetuximab is currently still recommended, mainly for the palliative treatment of recurrent and metastatic HNSCC [135,136], or R/M HNSCC. Cetuximab was further introduced as part of the EXTREME treatment regimen, combined with cisplatin or carboplatin, and 5-FU [137]. The EXTREME therapy still dominates first-line treatment of advanced cancers in many clinics. Furthermore, Cetuximab plus radiotherapy shows a surprisingly favourable toxicity profile and appears to be particularly active and beneficial in elderly patients that do not tolerate severely cytotoxic cisplatin treatment [136]. Additionally, a combination of Cetuximab with Cisplatin has shown moderate benefit in younger patients [138]. For the treatment of locoregionally advanced tumours, the current standard of care therapy is chemoradiation, combined with Cisplatin. Despite high drug costs and overall unsatisfactory response rates, Cetuximab has yet been evaluated as borderline cost-effective, compared to many other treatments [139]. Cetuximab treatment typically results in reduced angiogenesis or vascularization of the tumours (compare to Section 4), which points to another link to Notch signalling, in particular the NOTCH4/DLL4 axis. This is accompanied by characteristic response signatures, at the levels of miRNA and/or mRNA expression, that correlate with the simultaneous activity of Notch signalling [140]. At the same time, Cetuximab- responsive tumours showed upregulation of markers characteristic for Notch and stem cell signalling [141]. The same group has shown that treatment with EGFR inhibitors such as Cetuximab can also induce serious adverse effects. Cetuximab can induce proliferation of CAFs, or promote partial EMT and stemness in the tumour cells—all characteristics of active Notch signalling [141]. This finding, if corroborated in the clinics, may also affect the level of safety assigned to the clinical use of cetuximab, and probably other EGFR inhibitors as well. If Notch signalling is indeed functionally relevant for such adverse events remains to be elucidated.

A number of additional anti-EGFR antibodies have been recently developed and clinically tested, such as Panitumumab [142,143,144], Nimotuzumab [145,146,147], and Sym004 [148,149]. The anti-EGFR antibody Panitumumab was shown moderate activity as a single agent in HNSCC patients, previously treated with platinum-based drugs [142]. The benefits of adding Nimotuzumab to combination therapy with cisplatin and radiotherapy have been reported in some HNSCC patients [146], and high HIF1α (hypoxia-inducible factor 1-alpha) expression may indicate likely benefit for such treatments [145]. Sym004 has been in development for at least 10 years, and represents a mix of two chimeric monoclonal antibodies [150]. Sym004 has also been tested with moderate to good activity against HNSCC [151].

The EGFR pathway is also a target for a broad panel of receptor tyrosine kinase inhibitors (**RTKIs**) such as **Afatinib**, **Allitinib**, **Dacomitinib**, **Erlotinib**, **Gefitinib**, **Lapatinib**, or **Sorafenib**. These are low-molecular weight molecules that bind to the intracellular protein domains of EGFR, where they block phosphorylation and therefore signal transduction [130]. In addition to EGFR, some RTKIs also target other members of the ERBB tyrosine kinase receptor superfamilies, such as ErbB2 (HER2), ErbB3 (HER3), and ErbB4 (HER4) [152]. Overexpression of some types of ERBB superfamily receptors in HNSCC encourages clinical RTKI testing. For example, Lapatinib is a dual tyrosine kinase inhibitor that interrupts both EGFR and ErbB2 [153]. Lapatinib was shown to be effective at least in HNSCC primary cell cultures, or colony-formation assays; and such as Cetuximab, the inhibitory functions of Lapatinib are enhanced in combination with cisplatin [153]. Nineteen percent of tumour samples from HNSCC patients are ErbB2 positive [154], favouring attempts to further use Lapatinib in studies. Additionally, Afatinib may show some utility in the treatment of relapsed or metastatic HNSCC [155]. A phase II clinical trial with 340 HNSCC patients showed that Afatinib significantly increased the chance for progression-free survival by 37%, compared to other standard-of-care drugs such as Methotrexate. Yet, there were no differences in overall survival (NCT01856478). The response of HNSCC patients to anti-EGFR antibodies varies and seems to be associated with a high degree of tumour heterogeneity. It is therefore common to combine different signalling inhibitors, such as with **Copanlisib** (a PI3Kinase inhibitor), with Cetuximab, in order to improve treatment effectiveness [156]. Essentially, this leads to a double hit within EGFR signalling. Clinically testing these drug combinations in HNSCC may be particularly promising. There are attempts to improve treatment strategies targeting EGFR. For example, Driehuis et al. described the efficiency of EGFR-targeted antibody and nanobody photosensitizer conjugates in patient-derived HNSCC organoids model [157]. Combinatorial treatments targeting both Notch and EGFR, or mechanisms downstream of EGFR, such as PIK3CA, may therefore be promising—but have not been clinically explored yet. Figure 6 summarizes some of the most relevant small molecule inhibitors used currently in clinical trials targeting HNSCC, and their potential connection with Notch signalling.

#### 2.5.2. Crosstalk between Notch and EGFR Signalling

There is mounting evidence that the puzzling observation of bimodal activity of Notch signalling in early versus late stage HNSCC may be connected to the functions of the EGFR pathway. EGFR and Notch signalling are apparently linked, and both are likely to mutually influence or modulate each other. Unfortunately, most of the relevant findings for this hypothesis have been explored in SCC of other localizations, such as skin [158,159], but not in HNSCC. For example, it was found that EGFR can in principle act as a negative regulator of canonical Notch activity [160], a mechanism that most likely involves mutational knock-out of P53 and drives differentiation in normal skin keratinocytes and skin cancers. Since P53 is mutated in the majority of HNSCC cases, P53 mutation status also appears to modulate the functional connection(s) between EGFR and Notch signalling, with marked but not fully understood effects on tumour angiogenesis, proliferation, and apoptosis. Other, older findings essentially confirm these observations, and strengthen the putative links between EGFR and Notch signalling [159]. This link may partly rely on CAFs, as potential mediators of signalling, thus pointing towards the TME as a key player. This raises the possibility that EGFR and Notch signalling may act independently and parallel to each other, potentially via the stroma. There is surprisingly little newer work corroborating these earlier findings, but novel insights (and renewed interest) into the intra-tumour heterogeneity of HNSCC may pave the way for novel, more precise experiments to answer these questions.

Furthermore, there is plenty of evidence that specifically PI3K-mTOR/AKT and Notch signalling pathways may be more or less directly linked in HNSCC [161,162]. Notch and EGFR signalling may be indirectly linked via epigenetic mechanisms, mediated by key epigenetic modifiers such as LSD1—which can potently upregulate and activate both PI3K- and Notch-dependent genes [162]. This has been also observed in other cancer types such as prostate carcinoma [163]. A significant part of the crosstalk between EGFR and Notch signalling may be explained by specific interactions via PI3Kinase activity: Functional repression of Notch signalling effectively activated oncogenic PI3Kinase and AKT activities [164]. This further supports the hypothesis that absence of Notch signalling might result in a paradoxical generation of oncogenic activities. This may occur, for example, via untethering the EGFR-PI3Kinase-AKT-mTOR axis; an important finding potentially relevant for our understanding of the role of deleterious Notch mutations in HNSCC, and the pleiotropic functions of the Notch pathways. Generally, targeting overexpressed and mutated PIK3CA by small molecule inhibitors shows promise for therapeutic purposes in many cancer types and subtypes. This may in fact depend on Notch activity, or be modulated by Notch.

Mounting data further reinforce the concept of a strong and complex association among the EGFR/HIF-1α/NOTCH axis in the development of HNSCC. This association may be highly relevant for both, the mechanisms of action of EGFR inhibitors such as Cetuximab—but also for targeting Notch directly. Consequently, Notch signalling may modulate some of the most relevant downstream molecular effects of Cetuximab therapy- related to altered angiogenesis. A strong positive correlation between EGFR expression and increased HIF-1α has been observed in human HNSCC tissues [165]. Wherever treatment of HNSCC tumour tissues with Cetuximab shows signs of efficacy, significantly reduced angiogenesis is observed in both patients’ tumours and in a mouse model for HNSCC. Cetuximab simultaneously suppressed HIF-1α expression, microvessel density, and Notch signalling activation [165] in these experiments. In short, it appears that tumour angiogenesis in HNSCC simultaneously involves both the HIF-1α and Notch1 pathways. Targeting both EGFR and Notch simultaneously may therefore have the potential to boost therapy-response to single-target inhibitors such as Cetuximab, at least in subgroups of patients that remain to be identified. To corroborate this functional connection, it was demonstrated that the signal transduction through EGFR/AKT/MAPK pathway is associated with both increased HIF-1 activity [166,167] and NOTCH1 signalling [165]. Similar to many other tumours, the HIF-1 transcription factor is also critical in HNSCC, and was demonstrated to drive the expression of genes related to the induction and progression of angiogenesis (see also section on angiogenesis and Notch). This hypoxia-related gene signature in tumour cells/tissues is simultaneously associated with the promotion of EMT, and promoting resistance to anti-cancer drugs and radiation [168,169,170]. For example, the presence of laryngeal cancer stem cells that overexpress *KRAS*, *HIF-1α*, and *VEGF-A* is associated with increased resistance of HNSCC to Cetuximab [171].

Cetuximab-induced drug resistance may be linked to Notch, EGFR and HIF-1α signalling, and the role of the TME in these processes is poorly understood. On the other hand, some HNSCC cell lines overexpressing HIF-1α are more sensitive to Cetuximab, an effect that appears to be strongly cell line-dependent [172] and may not translate to in vivo conditions in tumours. Again, there are no studies available based on tissues yet that could further clarify these questions. In breast cancer cells, HIF-1α increases the release of the Notch NICD by increasing the activity of γ-secretase [173]. Moreover, the cooperation of NICD and HIF-1α led to an increase in SNAIL expression and, consequently, the progression of EMT [173]. Given fundamental similarities and overlaps between epithelial cancer types such as breast and head and neck cancers, one would expect that targeting the EGFR/HIF-1/NOTCH axis. For example, the combination of GSIs and HIF-1α inhibitors with Cetuximab could significantly increase the efficiency of HNSCC treatment [170] (Figure 6). A deeper understanding of these potentially critical interactions, which appear deeply rooted in the TME and centred around Notch signalling, would clearly be beneficial—but likely requires advanced, complex model systems that allow us to mimic cell–cell and cell–matrix interactions in vitro. See also Figure 6 for an overview of the most relevant inhibitors in clinical trials for HNSCC.

### 2.6. Immuno-Oncology Drugs: Targeting the Immune Checkpoints

Immune checkpoint (IC) receptors (ICR) are a group of costimulatory molecules that are responsible for attenuating the activation of immune cells during processes such as wound healing and infection. The pivotal role of the ICs network is to help to maintain manageable immune homeostasis within body tissues, by suppressing excessive activation of the immune system. This, in turn, prevents excessive tissue damage, the uncontrolled, overboarding release of cytokines and chemokines (“cytokine storm”), and the development of autoimmune diseases. It is well established that disturbances in IC control are associated with the development of a variety of cancers, including HNSCC [174,175]. HNSCC cells very often overexpress PD-L1 (CD274; B7-H1), a transmembrane protein that suppresses immune systems via the interaction with T and B cells PD-1 receptor. The introduction of immune checkpoint inhibitors (ICI) has improved treatment options for common solid tumour entities, including HNSCC—although the benefit here is limited to approximately 20% of the patients that respond or partly respond. **Pembrolizumab**, [176,177,178,179,180], **Nivolumab [181,182,183,184]**, and **Sintilimab** [185], all inhibitors of the PD-1 receptor, expressed on the surface of (exhausted) T and B cells showed limited benefits in treating HNSCC.

The recombinant, fully humanized antibodies Nivolumab (Opdivo; [183,184]) and Pembrolizumab (Keytruda; [186]) Click or tap here to enter text.were first approved in 2016 for the treatment of patients with platin-refractory recurrent and/or metastatic HNSCC. Both Nivolumab and Pembrolizumab were first clinically tested [187] against the now firmly established EXTREME regimen, but have not significantly changed patient outcome in clinical practice. The overall response rates are only modest, although durable tumour regression has sometimes been observed even in refractory cases with platinum resistance [188]. Since 2019, both checkpoint inhibitors have been evaluated as safe [178,189,190] and are now approved for first-line treatment of primary tumours. Response rates to Pembrolizumab in these clinical trials for patients with HNSCC range between only 15–20% but may increase to approximately 30% when combined with standard-of-care treatment (SOC) drugs such as cisplatin and 5-FU. Yet, the largest fraction of ICI-treated patients in HNSCC are non-responders; while others (around 16%) suffer from adverse effects or hyper progression [191]. Similarly, the recombinant antibody Nivolumab has been tested and approved for the treatment of HNSCC patients [184,189] and found safe and at least partly effective [190,192]. Unfortunately, also Nivolumab showed very differential efficacy against primary tumours vs. metastatic lesions [192]. The most recent results of large multi-centre trials are reported in [193]. Nivolumab and Pembrolizumab can be considered as therapeutic alternatives, but both represent costly therapies. In several independent studies, these novel drugs were evaluated as only borderline cost-effective, primarily due to very high drug prices [181,194,195,196,197] of > USD 100,000 per patient/year. Generally, the incomplete and unsatisfactory response rate against both antibodies (and similarly, the poor response against Cetuximab) requires additional fundamental research into the mechanisms of primary and secondary (acquired) drug resistance, and specifically the role of the immune-suppressive TME in those 70% of cancer patients that show no response, or even adverse events (hyper progression).

Combination therapies of ICI with other, standard-of-care drugs may improve efficacy, for example in combination with cisplatin [198,199]. This may also apply for combining ICIs with different targets: Nivolumab is currently being tested in combination with **Ipilimumab** targeting CTLA4 [200]. Results of this trial are pending (NCT03700905 ClinicalTrials.gov, last accessed Nov. 17, 2021). Next, combination-therapy of Pembrolizumab with standard-of-care chemotherapy versus Cetuximab (targeting EGFR) was further tested in the KEYNOTE-048 and KEYNOTE-040 clinical trials [136,176,177,201] and also considered safe [202], but again showed significant survival benefits in only some patients [198]. The combination of Pembrolizumab with other therapies, such as chemoradiation, or as a maintenance therapy, was tested in the KEYNOTE-412 trial [165,203]. Many other drug combinations are currently being tested, for example with the epigenetic HDAC inhibitor Vorinostat [204]. 

In this context, it may also be interesting to investigate whether Notch signalling activity may modulate the patients’ response to immune checkpoint inhibitors. This is an emerging field of research, with so far limiting insights; nevertheless, these are indicating that there likely is an interference. There have been no specific studies investigating the impact of Notch signalling, or Notch pathway mutations, on outcome and response of patients to ICIs in HNSCC. The only experimental study based in HNSCC cell lines dates from 2018 [205]. Treating HNSCC cells with the γ-secretase inhibitors GSI-IX and DAPT decreased tumour burden in a genetically engineered mouse model. Detailed analyses showed that inhibition of Notch signalling reduced the cell numbers of myeloid-derived suppressor cells (MDSCs), tumour-associated macrophages (TAMs) and regulatory T cells (Tregs) within the emerging tumour tissues. In addition, inhibition of Notch signalling significantly inhibited mRNA and protein expression of the most relevant immune checkpoint molecules (PD1, CTLA4, TIM3 and LAG3), all of which represent targets for approved or developmental ICIs. In contrast, elevation of elevation of the NOTCH1 downstream target HES1 correlated significantly with increased numbers of myeloid-derived stem cells (MDSC), TAMs, and T-regulatory cells (Treg) in the mouse cancer tissues [205].

However, a growing amount of evidence has become available from studies of non-small cell lung cancer (NSCLC), including squamous cell carcinomas of the lung (LSCC). NOTCH mutation in NSCLC might be a robust predictor of immunotherapeutic efficacy. A bioinformatic study, for example, revealed striking correlation between mutations in NOTCH1-3 receptors, and better outcome to ICI therapy [206]. NOTCH mutation status, especially deletions, was independently associated with immunotherapeutic benefit by ICI therapy. The authors suggested that the presence of Notch mutations could serve as a potential predictor for favourable ICI response in NSCLC. Furthermore, it was insinuated that Notch mutation or -deletion status may serve as an important predictor for personalized combination immunotherapy, and that combining NOTCH inhibitors such as GSI with ICI therapies may show synergistic or combinatorial benefits for the patients [206]. Another recent study based on NSCLCs also showed that high frequency mutations in Notch-pathway related genes could serve as an independent predictor for NSCLC patients receiving ICI therapies [207]. Highly mutated NOTCH signalling strongly correlated with the inflammatory immune microenvironment, an inflammatory mRNA gene expression profile, and enhanced immunogenicity. Yet, another recent study in lung cancers essentially confirms these findings [208]. High frequency of mutations in either the NOTCH receptors 1-3, or in homologous DNA mismatch repair (HR) genes were associated with improved therapy outcome and longer survival in NSCLC patients treated with ICIs. The authors conclude that mutations occurring in both the NOTCH and the HR pathways may be functionally associated with increased efficacy of immune checkpoint blockades in advanced NSCLC. A recent follow-up study [209] has essentially confirmed the findings of the earlier paper from 2020. The authors were able to detect detected alterations in the NOTCH pathway in 71% patients that showed durable clinical benefit (DCB) to immune checkpoint inhibition. In contrast, only 36% of the patients that did not respond to ICIs with durable benefits. A similar association was observed for DDR or DNA damage response genes.

Naturally, it remains open if similar observations related to Notch signalling and immune checkpoint therapies are also valid in HNSCC.

Another source of information that may be also relevant for HNSCC are gastric cancers. Expression and activity of NOTCH3 was identified as a good prognostic factor for immunotherapy of gastric cancer patients [210]. High levels of *NOTCH3* expression correlated with significantly reduced infiltration of activated CD8^+^ T cells in the cancer tissues. In contrast, high NOTCH3 was associated with increased infiltration of M2 macrophages and Tregs into the TME, both immunosuppressive cells. Last not least, the authors also observed increased expression of immune checkpoint inhibitors, resulting in suppression of immune response. High NOTCH3 expression further negatively correlated with other, established and widely used predictive biomarkers for the success of ICIs, including high tumour mutation burden (TMB) and the innate anti-PD-1 resistance (IPRES) signature.

Furthermore, *NOTCH4* mutations have been identified as a putative biomarker for poor response to checkpoint inhibitors [211]. Bioinformatic tools such as the use of immune-related gene pair (IRGPs) signatures allows predicting the prognostic biomarkers for HNSCC patients for further immunotherapy, consequently resulting in higher treatment efficacy and lower drug toxicity [212], and this may well correlate with Notch signalling activities in the tissues. For example, during the activation of CD8^+^ T cells, NOTCH1 signalling leads to an increase in PD-1 expression [213], which consequently increases the sensitivity of T cells to suppressive signals from the TME. Additionally, it has been shown that inhibition of NOTCH1 signalling can lower PD-1 expression [214], which could be used for therapeutic purposes. Interestingly, HNSCC HPV-negative patients undergoing anti-PD-1/L1 therapy have more frequent mutations and frameshift events of the *NOTCH1* gene [215].

Similarly, **Durvalumab** [216,217,218,219], also known as MEDI4736, blocks PD-1 and CD80 (B7-1) from binding to cell expressing PD-L1. **Ipilimumab** [220] and **Tremelimumab** [217,219,221,222] both target the receptor CTLA4 and consequently block inhibitory signals from CD80 and CD86 (B7-2) co-stimulatory proteins. Data from a meta-analysis showed that neoadjuvant chemotherapy by PD-1 and PD-L1 ICIs may be well tolerated and effective for HNSCC patients [223], which encourages further research. However, some side effects of ICs inhibitors related to systemic organ damage via over-activation of the immune system need to be considered [59].

**Mogamulizumab**, an inhibitor of the CCR4 chemokine receptor expressed on T-regulatory or Treg cells, may be used to reduce the Treg-dependent suppression of immunity. Inhibition of Treg by Mogamulizumab has been suggested to interact with PD-1 blockade to enhance anti-tumour immunity. Combination therapy with **Mogamulizumab** and **Nivolumab** showed anti-tumour activity against solid tumours [224]. However, in combination therapy with **Durvalumab** or **Tremelimumab**, administration of **Mogamulizumab** did not increase the antitumour effect of both antibodies in case HNSCC patients [225]. Unsatisfactory results were also obtained during trials with the use of **Cixutumumab** (IMC-A12), an IGF-1R (insulin-like growth factor-1 receptor) inhibitor [226]. Several novel recombinant antibodies have been designed to act as an immune receptor agonist to stimulate an immune response. **Utomilumab** and **Urelumab** (PF-05082566) bind to the 4-1BB receptor (CD-137) presented on CD8^+^ and CD4^+^ T cells and NK cells, consequently stimulating their activity and increasing proliferation [227] currently also being tested against HNSCC [228,229]. Figure 6 also summarizes some of these drugs, currently in clinical trials.

### 2.7. Cell Cycle Inhibition and Notch Signalling

HNSCC is characterized by strong intracellular signalling related to the processes of intense proliferation, growth, and cell division. Therefore, it may not be surprising that some of the newer, second-generation Notch inhibitors such as FLI-06 (already mentioned in Section 2.4) appear to significantly affect cell proliferation, or may even partly act via inhibition of growth and proliferation [116]. FLI-06 appears to inhibit Notch signalling by blocking the expressions of NOTCH3, and downstream target genes *DTX1* and *Hes1*. It also affects the expression of histone lysine specific demethylase LSD1, again pointing to a potential link between Notch and LSD1 activities [162,163]. Otherwise, there are surprisingly few studies focusing on any possible interference, synergism, or just added effects between blocking Notch signalling, and the plethora of anti-cancer drugs targeting cell cycle progression and mitosis in HNSCC.

Some of the newer and ongoing developments are summarized below. **Rapamycin** (known as Sirolimus; NCT01195922) and the Rapamycin ester, **Temsirolimus**, are inhibitors of the mTOR (mammalian target of Rapamycin) complex, which cause cell arrest in the G_1_ phase of the cell cycle and consequently reduces cell proliferation [230]. Especially Temsirolimus shows promising anti-tumour activity in HNSCC cell lines with low EGFR expression [231] and also in combination with Carboplatin and Paclitaxel [232], or Bevacizumab and Cetuximab [233]. These variable and often synergistic drug combinations could be a solution for further treatment of advanced, metastatic head and neck cancers (M/R HNSCC). Similarly, **Palbociclib**, a selective inhibitor of the cyclin-dependent kinases 4 and 6 (CDK4/6), leads to inhibition of head and neck tumour cell proliferation by inducing cell cycle arrest. Palbociclib may be effectively combined with Cetuximab (NCT03194373), [234,235]. In addition, Palbociclib increases the sensitivity of HPV^-^, but not HPV^+^ HNSCC cell lines to the inhibitor of the BCL2 protein family, **Navitoclax** [236]. However, some data indicate that prior exposure to Cisplatin can significantly reduce Palbociclib activity [237]. **Methotrexate** (MTX), an antimetabolite of folic acid that inhibits DNA replication during the S phase of the cell cycle, is also being studied against HNSCC with moderate success [238,239]. The siRNA profiling of HNSCC primary HPV^-^, p53 mutant cells, indicates their sensitivity to agents targeting the G_2_-M phase of the cell cycle [240]. **Vinflunine ditartrate**, a fluorinated microtubule inhibitor belonging to the Vinca alkaloids family, stops microtubule polymerization during the metaphase/anaphase transition of the G_2_-M phase of the cell–cell cycle [241]. Due to insufficient results on activity against other types of cancer, Vinflunine ditartrate is currently in Phase III clinical trials for metastatic HNSCC (NCT02347332).

**Alisertib** (MLN8237) [242,243], a selective inhibitor of the G_2_-M phase-activated Aurora A kinase and **Ispinesib** [244], a selective inhibitor of the kinesin spindle protein (KSP), which control spindle dynamics during mitosis, were also tested against HNSCC. **Prexasertib**, an inhibitor of CHK1/2 proteins related to control of cell cycle and DNA repair processes, increases the sensitivity of HNSCC cells to Cisplatin and radiation, which in turn leads to an increase in cell apoptosis, and these actions were accompanied by inhibition of NOTCH signalling [118]. These examples only represent a relatively small part of the current developments and are probably not representative of the entire field.

## 3. Notch Signalling and Tumour Cell Plasticity

### 3.1. Tumour Cell Plasticity as a (Moving) Target

Temporary and dynamic changes at molecular and phenotypic levels without genetic mutations, especially in response to environmental cues, are collectively referred to as cellular plasticity. Increased (tumour-)cell plasticity often characterizes pathological states and accompanies changes that neoplastic cells undergo during tumour progression [245]. When epithelial tumours or carcinomas progress, significant changes in cellular identity or plasticity in the now transformed cells occur regularly. This involved a spectrum of genetic, and non-genetic changes that are characteristic for different stages of tumour progression. These are often associated with equally characteristic morphological or phenotypic changes in the corresponding tumour tissues. The most relevant processes related to, or driven by, enhanced tumour cell plasticity are: (1) **epithelial-to-mesenchymal transition (EMT)** and (2) the development of increased **tumour stem cell characteristics** or **stemness**. Stemness as the result of plasticity is ultimately thought to lead to the development and selection of cancer stem cells (CSCs) that show reduced proliferative potential, but simultaneously increased therapy resistance. A third process (3) is enhanced cancer cell motility, or **invasiveness**, which is considered a main factor for aggressive tumour growth, penetration of blood and lymph vessels (intravasation/extravasation and vascular mimicry), and various forms of local lymph node or distant metastasis. These processes may in fact represent a phenotypic continuum that benefits from increased tumour cell plasticity. Increased plasticity may predominantly be a sign of reduced cellular commitment to terminal, functional differentiation, or lineage-specific maturation of tumour cells—replaced by enhanced cellular freedom to operate. Loosely speaking, enhanced plasticity provides transformed cancer cells with the phenotypic fluidity that is required to behave as partly anarchic mavericks within living tissues, ignore or transmutate differentiation signals. We will also investigate how these three processes may be driven or modulated by altered cell signalling, especially in HNSCC.

### 3.2. Introducing EMT as a Hallmark of Enhanced Tumour Cell Plasticity

In oncology, EMT and its reversal, **mesenchymal-to-epithelial transition** (MET), have long been studied as (potentially) critical aspects for tumour initiation, progression, aggressive invasion, and metastasis. The EMT/MET continuum was repeatedly shown as a prerequisite for—or at least promoting—acquired resistance to chemo- and/or radiotherapies. Exploring and defining EMT during tumour progression appeared, for a long time, to be the perfect model to study the nature of tumour cell plasticity [246]. However, it is still puzzling what may be the role of EMT and EMT-like processes in cancer progression, versus normal differentiation processes. Currently, it appears that EMT may promote the general aptitude of tumour cells towards rapid phenotypic changes or be the result of these: cause and effect are not clearly defined. The invasive spread of cancer cells is only one possible phenotype, and maybe only the most outstanding feature supported by EMT. Generally enhanced tumour cell plasticity could represent a key hallmark of advanced tumour cells. This remarkable spectrum of phenotypic fluidity, of which EMT is only one result, is probably based on the continued need of tumour cells for strong rapid selective adaptation to changing environments and stress conditions. It may be highly beneficial for tumour cells simply to stay poised for rapid reactions to almost any incoming environmental cues. At the same time, this high degree of adaptability also integrates potent survival signals that are provided by cell–cell contacts, cell–cell, and cell–matrix communication, not from invasive motility. Epithelial integrity therefore continues to be crucial for tumour cell growth and survival. Even though more mobile, EMT-associated phenotypes may facilitate initial dissemination, survival and continued growth and proliferation are as important for successful cancer progression, and survival of tumour cells. This is particularly critical for tumour cells actively penetrating or populating distant metastatic sites, also known as colonization [247]. Not surprisingly, the aspect of continued epithelial “squamousness”, may remain a very dominant feature that is rarely completely abandoned [7], even by successfully invading and highly metastatic tumour cells. As a result, even advanced and aggressive epithelial cancer cell prefer collective over amoeboid invasion modes, in which cell–cell-contacts are retained, despite enhanced tumour cell motility. A high degree of plasticity may be critical to adapt to environmental changes, including the exorbitant stress that comes with radio- and/or chemotherapy, or resisting the continuous onslaught of the patients’ own immune system [248]. Plasticity and the EMT/MET continuum also helps cells that are constantly traveling within and between different conditions of the ECM which is associated with variable stiffness, rigidity and density, and shifting access to signalling molecules [249,250]. A highly dynamic EMT/MET continuum of plasticity (see Figure 7 and graphical abstract) may also help tumour cells with penetrating the basement membrane of epithelial tissues, blood and lymph vessels; including intravasation and extravasation, or surviving in the hostile and very different environment of lymph nodes and the blood stream [251]. The EMT/MET continuum depends on the induction of transcriptional programs, and specific transcription regulatory factors that alter gene expression patterns, to initiate and promote the loss of cell–cell adhesion. Most prominently, this leads to a shift in cytoskeletal dynamics and a change to the more mesenchymal, intrinsically more motile phenotype. This switching ON and OFF of specific transcription programs in EMT, thus enhancing plasticity, is induced by signalling pathways mediated by a spectrum of external factors [252]. These are, for example, signalling through transforming growth factor beta (TGF-β), [250] and bone morphogenetic protein (BMP), [249], Wnt-β-catenin, Hedgehog signalling [253], and diverse receptor tyrosine kinases, including EGFR and FGFRs.

EMT is a physiological process related to embryonic morphogenesis, the formation of foetal tissues and tissue regeneration, or wound healing [254]. However, EMT also occurs or is hijacked in pathological processes such as fibrosis [255,256], cancer initiation and tumour progression. EMT is characterized, in practice, by reduced expression of the “classic” epithelial marker E-cadherin, which allows for the (partial) loss of cell–cell junctions and epithelial integrity of the tissue. In epithelial cancers or carcinomas, and even more prominently in squamous cell carcinomas such as HNSCC, this leads to a (partial) loss of epithelial orientation of apical-basal polarization, or maturation [257]. Characteristic for EMT is further the loss of epithelial markers such as cytokeratins 8 and 18, and the over- or de novo-expression of mesenchymal markers such as vimentin and N-cadherin. This is often (but not always) accompanied by high expression of stem-cell markers (CD44, SOX2, Nanog, ALDH1, etc.). Functionally critical is the expression of transcriptional regulators such as the **E-box binding transcriptional repressors** such as Snail and Slug (SNAI1 and SNAI2), TWIST1 and 2, or ZEB1 and ZEB2, further supported by increased expression of miRNAs of the miR200 family [258]. We will see further down in this chapter that also miRNAs expression networks are highly relevant for EMT and fine-tuning tumour cell plasticity. As a result, the less organized tumour structures create a local microenvironment conducive for increased cell mobility, which is thought to promote invasion and metastases [259]. Thus, EMT not only plays a key role in the generation, selection, and survival of tumour cells, it is simultaneously the product of enhanced mesenchymal or stromal-cell-like properties, and phenotypic heterogeneity [260,261].

In clinical practice, prominent EMT-related characteristics—originally identified in cancer cell lines—have traditionally been loosely associated with phenotypic changes in tumour biopsies—which we have encountered as features relevant for patient stratification and prediction, such as “tumour budding”. These histopathologic phenotypes may be defined or shaped by enhanced cell motility, thus potentially promoting local invasion [262,263] within the tissue. Active EMT within living cancer tissues simultaneously promotes cancer stem cell properties, or stemness, further continuing down the spectrum of tumour cell plasticity or phenotypic heterogeneity in tumour tissues [261,264]. EMT in cancer tissues may also be involved in lymph angiogenesis (invasion of lymph and blood vessels), and the formation of local or lymph node metastasis [265]. The translation of in vitro findings to clinical settings however remains challenging, especially for dynamic processes such as EMT. EMT is further suspected (but not strictly proven) as one of the main factors associated with secondary, or acquired resistance to therapies [251,253,259,266]. Accordingly, histologists showing enhanced EMT-like characteristics have been statistically correlated with patients that show poor outcome—and develop resistance to standard-of-care chemotherapeutics such as Cisplatin [267] or anti-EGFR therapeutics such as Cetuximab [172].

### 3.3. Visible versus Hidden Forms of EMT in HNSCC

One unresolved issue in HNSCC is therefore the question: How do we recognize active EMT or hybrid-EMT in cancer tissues? Additionally, what are the consequences? Histological features such as “tumour budding” may be a strong sign of an active EMT in more aggressive tissues, and the may also correlate with poor therapy response and patient outcome [268,269]. However, HNSCC biopsies only occasionally show strong expression of characteristic mesenchymal markers such as vimentin or N-cadherin, or loss of cytokeratin 8 and 18 expression, for example at the invasive front. Thus far, no clear, characteristic and reproducible differences in EMT-related biomarker expression have been functionally validated in tumour cells and tissues with or without lymph node metastases [257,270]. It is still debated whether EMT indeed correlates or pre-disposes to recurrence and distant metastases [271,272], or if these are completely independent processes. The initial enthusiasm of the research field concerning EMT as a deceivingly simple explanation for aggressive tumour cell behaviour has therefore not been fulfilled. To a large degree, EMT may go invisible and unnoticed by clinical pathologists, also due to the static nature of formalin-fixed, paraffin-embedded tumour tissues. At the same time, there are few experimental models that allow conclusions from experimental work with isolated cancer cells for clinical purposes. The emerging topic of EMT, especially in HNSCC, has recently been reviewed in numerous excellent articles, such as [253,273,274]. Naturally, some of the reviews also touch upon the impact of Notch signalling on the execution and regulation of EMT-like processes, but there are none that specifically focus on the issue.

### 3.4. Hybrid or Partial EMT in HNSSC and Other Epithelial Cancers

A more critical and differentiated point of view of EMT has only been emerging in recent years—the concept of **partial or hybrid EMT**, often abbreviated as “E/M” state. This E/M state is characterized by the simultaneous presence of both epithelial and mesenchymal features [274], and an incomplete or partial expression of the characteristic panels of mesenchymal biomarkers. Hybrid or partial EMT cells have been further shown to possess greater tumour initiation potential and resistance to treatment, than epithelial or mesenchymal cells [264,275]. Moreover, the partial EMT profiles may vary, even within one and the same tumour biopsy, which contributes to generating a very high level of intra-tumour heterogeneity [274]. Undergoing a partial or hybrid EMT may come almost automatically with the return ticket: the option to also undergo an MET, especially if the TME and ECM promote this decision [250]. This generalized process of enhanced tumour cell plasticity simultaneously has the potential to generate increasingly motile cells with mixed, mesenchymal and stem-cell-like properties [257,261,271,274]. Enhanced plasticity then leads to partial loss of the characteristic lineage-specific epithelial differentiation, or maturation. Unfortunately, the highly dynamic nature of partial- or hybrid EMT processes has so far precluded this process from being directly targeted in cancer by specific, pharmacological inhibitors. This is the point where Notch signalling may enter the discussion: Notch is one of the central pathways that regulate tissue formation, integrity and maintenance (homeostasis), steering lineage-specific differentiation versus stemness. More importantly, Notch signalling may represent—in contrast to a fluid process such as EMT—a valid molecular target for chemotherapies.

Only recently, single-cell RNA sequencing studies (scRNA-Seq) have shed some more light on the true nature of EMT-like processes or enhanced tumour cell plasticity in human HNSCC tissues. They have essentially confirmed (although with a twist), that aggressive tumour cells can indeed assume a transient differentiation state. This represents a typical example of hybrid or partial EMT [276,277]. These scRNA-Seq studies [46,278] have further revealed that only a subset of transformed tumour cells embedded within the HNSCC tissues display the partial-EMT or E/M program, for example on the surface of larger “tumour islands” embedded in the stroma. These EMT-like cells express large amounts of mRNAs for extracellular matrix proteins. Furthermore, the EMT-like tumour cells are localized directly at the leading edge of primary tumours, in very close proximity to cancer-associated fibroblasts (CAFs) and/or myofibroblasts. The similar morphology of both cell types has been confusing pathologists, not being able to distinguish between transformed, squamous carcinoma cells that acquire mesenchymal-like phenotypes (or histology) by a partial EMT, and true stromal fibroblasts, that have retained the original mesenchymal differentiation and phenotype. It is now clear that E/M tumour cells are definitely of epithelial origin, and just display variable morphologic features of mesenchymal cells. Furthermore, scRNA-Seq analyses also verified that these mesenchymal-looking, true tumour cells surprisingly lack expression of classical EMT transcription factors such as Snail, Slug and Twist. It also appears that the numbers of CAFs versus E/M cells is highly variable between patients, and even within the same biopsy. This extraordinarily high degree of intra-tumour heterogeneity has led histopathologists to misclassify CAF-enriched tumours into a distinctive, but erroneous, tumour subtype [46], which had been previously classified “mesenchymal subtype”. Puram et al. have now convincingly shown that this “stromal-like” tumour subtype in fact corresponds to malignant basal-like tumours.

### 3.5. Regulation of EMT in HNSCC by Notch Signalling

Upregulation of the Notch pathway has been shown to occur in many HNSCC patients’ tumours, and *NOTCH* expression has been positively associated with an advanced clinical stage [279], poor overall survival, enhanced number of positive lymph nodes and distant metastases, and many other predictive aspects including EMT. Thus, it has been assumed for at least 2 decades that inhibition of Notch signalling may represent a druggable, therapeutic modality—but until today, this has remained an elusive goal. Nevertheless, the depletion of *NOTCH1* in HNSCC cell lines resulted, not surprisingly, in down-regulation of Notch-target genes such as *c-Myc*, and to reduced EMT-like phenotypes, such as lower tumour cell motility and invasion [280].

Some of the most relevant consequences of activating canonical Notch signalling have been functionally validated in this way, but only in vitro. Notch promotes invasion, migration, and metastasis in different types of solid tumours [259,281], which are naturally correlated with a partial/hybrid EMT. Due to its ubiquitous nature and crosstalk with other signalling pathways, the Notch signalling pathway can also influence other, functionally related processes that enhance and maintain the interim-differentiation state defined by an EMT [282]. For example, it was shown that inactivation of Notch signalling by GSI could reverse the EMT process in HNSCC and oral squamous cell carcinoma (OSCC) [280,283]. Some of these Notch-related effects have yet to be addressed in a broader context, including the cell–cell-interactions with other cell types in the TME, and also related to the composition, density and stiffness of the ECM. Nevertheless, it becomes rather apparent that Notch signalling may play an important role in regulation of EMT in HNSCC. The question remains, how this occurs; and how this is relevant for clinical cancer tissues.

There are several reports illustrating that NOTCH1 expression (mRNA and protein levels) is elevated in tongue cancer tissues, and *NOTCH1* expression was associated with tumour stage and de-differentiation, or therapy resistance [284]. Some recent studies showed that overexpression of *NOTCH1* can increase proliferation, invasion and migration of tongue cancer cells, and inhibition of *NOTCH1* expression reverses these processes and instead promotes apoptosis in vitro and in vivo [285]. Similarly, in tongue squamous cell carcinoma (TSCC) tissues, overexpression of NOTCH2 was linked to increased proliferation, migration, and invasion abilities of cancer cells [109]. In laryngeal squamous cell carcinoma, active NOTCH2 signalling was linked to lymph node metastasis [108]. In striking contrast, it has been shown that NOTCH3 can significantly weaken the EMT process in HNSCC cells [270]. *NOTCH3* overexpression in oesophageal squamous cell carcinoma (ESCC) cells not only suppressed EMT, but also sensitized these cells to therapy—suggesting striking differences in the physiological roles of the NOTCH receptors. As a logical reversal of this signalling, it was shown that silencing of *NOTCH3* promotes EMT and increases chemoresistance of HNSCC cells [100,114]. Furthermore, high expression levels of *NOTCH4* appeared to promote EMT and plasticity. *NOTCH4* showed a significant correlation with overexpression of specific mesenchymal markers, such as *N-cadherin (CDH2), Vimentin, TWIST1, SOX2*, *Fibronectin*, and downregulation of *E-cadherin (CDH1)* in patients with HNSCC. As expected, silencing of *NOTCH4* by *siRNA* in the same HNSCC cell lines significantly increased the expression of epithelial marker such as *CDH1*, and decreased the expression of mesenchymal markers [115,281]. In HNSCC and OSCC cells, high levels of expression and activity of NOTCH4 was significantly related to increased proliferation, chemotherapy resistance, cell cycle, apoptosis inhibition, and EMT. This was confirmed by genome-wide data mining, using the genome-wide TCGA data sets, and further validated in vitro [281,286]. The NOTCH4-HEY1 pathway may specifically promote EMT and may also lead to increased invasion and migration in HNSCC [270]. Although the mechanism of action of all NOTCH receptors (1–4) has been thought to be essentially identical, or highly similar, the studies mentioned above all suggest that NOTCH target genes and the impact for affected cells are likely to be different from the specific receptor that is engaged. There may also be pleiotropic effects of other NOTCH signalling elements on the EMT phenotype.

### 3.6. How NOTCH and EMT Signalling May Be Connected

A growing number of studies support the idea that Notch signalling is one of the key pathways related to the control of the EMT process. The details in how exactly this is achieved, however, are only slowly developing. Some current reports rather support the hypothesis that activation of NOTCH1 in HNSCC only recapitulates its usual biological function which is to regulate a program of gene expression associated with very early differentiation, or transit amplifying cells, committed to undergo epithelial (or squamous) differentiation, rather than having a strong impact on EMT or cancer stem cell maintenance (reviewed in [88]). This topic remains controversial and also applies to cancer stem cell biology (see below). One of the most relevant areas of research have investigated the interaction between Notch signalling, and the SNAIL/SLUG transcriptional regulators, or E-box proteins, whose genes are targeted by the NICD. The perhaps most relevant transcriptional regulators involved with driving an EMT in squamous carcinoma cells are the SNAIL factors, which are transcriptional repressors that bind specifically to the *E-box* motif, such as at the *CDH1* promoter, thus inhibiting its expression [287]. In return, high activity of SNAIL transcriptional regulators in epithelial cells leads to an increase in the expression of mesenchymal factors such as *CDH2* or *vimentin*, well-known markers of the EMT process [274]. Studies on HNSCC cell lines have shown that EMT induced by overexpression of the *SNAIL* transcription factor led to increased drug resistance, invasiveness, and metastasis [288]. Snail-induced EMT also supports a stem-cell-like phenotype and enhances spheroid formation ability, chemoresistance and invasive capacity of HNSCC cells [288]. Interestingly, under hypoxic conditions in which hypoxia-induced factor 1α (HIF-1α) binds to CSL, NICD, and MAML, higher expression of *SNAIL*, *NOTCH* receptors, and ligands was observed [289], suggesting an important role of hypoxia/NOTCH/EMT axis in the development of HNSCC. Several studies further suggest that the SNAIL transcription regulator may be one of the key molecular targets in developing therapeutic strategies for HNSCC.

SLUG, a related transcriptional repressor belonging to the same family of transcription repressors as SNAIL, performs a similar function in NOTCH-related EMT progression [287]. Moreover, it has been shown that SLUG can serve as a surrogate marker for probing EMT in head and neck cancer cells [290]. A number of reports have shown the potential link between SLUG activities [290], and formation of an EMT-like phenotype, including histologic features, and the expression of EMT markers in HNSCC tissue samples. Interestingly, the authors found that a putative EMT-like process in cancer tissues can be assessed in a straightforward fashion by staining for cytokeratin/vimentin co-expression, and the simultaneous loss of E-cadherin/β-catenin co-expression. SLUG was further identified as a potential surrogate marker for EMT-like processes in tissues. HNSCC progression in patients may be closely related to EMT, induced or promoted by the activation of the Notch pathway in late-stage, aggressive HNSCC tumours.

Another NOTCH-regulated, EMT controlling transcription factor related to HNSCC progression is ZEB1 (zinc finger E-box-binding homeobox 1). It has been shown that ZEB1 inhibits the expression of cellular polarization factors, which is crucial for the maintenance of the epithelial phenotype. CD133 (^+^) CSC-like cells from head and neck cancer patients showed a markedly increased expression of both, *ZEB1* and *ZEB2* factors. Silencing these factors influenced the ability to self-renewal, drug resistance, and expression of stemness markers [265]. Additionally, in the presence of TGFβ in the TME, NOTCH1 and ZEB1 cooperate and jointly cause inhibition of *NOTCH3* expression, which consequently promotes tumour initiation and EMT [270]. Additionally, some additional mesenchymal transcriptional regulators may be capable of further enhancing Notch signalling. A good example for this class of factors is TWIST1, which activates Notch by enhancing the Notch-downstream genes, such as *HES1*, *HEY1*, and *HEY2*. However, no increased expression of TWIST1-dependent Notch ligands nor NOTCH1 was observed in oesophageal squamous cell carcinoma patients [291].

Apart from NOTCH signalling, also several other pathways may affect epithelial differentiation and are themselves modulated by the HPV oncogenes E6 and E7. The interaction of E6 and E/ with cell cycle regulatory proteins was mentioned in chapter 1, but also other HPV oncoproteins may have an impact. For example, HPV5 E6 was able to impede TGFβ signalling (and thus may influence EMT processes), by binding to the SMAD3 transcription factor, possibly disrupting both SMAD3 and SMAD4 signalling. The HPV5 E6 oncogene, but not E7, was shown to suppress a SMAD-dependent luciferase reporter, downstream of the TGFβ receptors. Additionally, the beta-type HPV E6 proteins were shown to interfere with the formation of a SMAD-containing transcriptional activator complex at the p15INK4B (*CDKN2B*) promoter, for example after TGFβ treatment. In comparison, high-risk HPV E7 oncoproteins can alter the levels of *p63*, via binding to the central retinoblastoma protein (Rb), a master regulator of epidermal development in epithelial cells. The intracellular P63 protein levels and transcriptional activities are typically reduced upon epithelial differentiation, and targeting *p63* (among many other genes) may specifically assist HPV-mediated changes in squamous differentiation versus continued proliferation. Tight regulation of squamous differentiation is also essential for effective viral reproduction of all HPV types in normal mucosa but is typically disturbed by genetic alterations (deletions), or insertion of the viral DNA into the host genome, in transformed carcinoma cells. The presence of HPV31, and specifically the high-risk HPV31 E7 oncoprotein, were shown to effectively decrease P63 levels, which is in line with the need to disturb these differentiation-related processes, and interfere with lineage-specific decisions. The same is observed by an upstream regulator of P63 expression, the miRNA miR-203. In HPV-positive keratinocytes, levels of P63, and other cell cycle regulatory proteins, such as cyclin A, cyclin B1, CDK1, and CDC25c, were all decreased, and late viral activities were typically damaged or defective. Finally, it was found that the degradation of the PTPN14 protein represents a critical, retinoblastoma-independent function of high-risk HPV E7 oncoproteins. The effect of PTPN14 degradation was shown to restrict differentiation, an activity that is crucial for HPV-mediated oncogenic transformation [292].

### 3.7. EMT Regulation within the TME

The TME has a strong and often dominant influence on the fate of cells, including on EMT and cell plasticity. These associations have often been neglected since the transformed tumour cells themselves have been almost entirely in the centre of attention of research. Nevertheless, many tumour-associated cells, such as fibroblasts, immune cells, endothelial cells in capillaries and blood vessels, express both Notch ligands and receptors simultaneously. This is relatively well investigated in HNSCC; in particular the expression of Jagged1 and -2 in endothelial cells/capillaries within the tumour tissue [293,294], or of DLL4 [35,295,296]. These cell–cell interactions specifically activate Notch signalling pathways through direct mechanical contact, mostly by heterotypic or trans-signalling. This, in turn, is likely to drive EMT progression by NOTCH receptors and their ligands interactions and can trigger a cascade of reactions in neighbouring cells, thereby radically changing their fate. For example, tumour cells that are engaged in partial/hybrid EMT, driven mainly by Notch-DLL interactions, typically remain largely epithelial, and tend not to become overtly invasive. Simultaneously, this local signalling may promote tumour cell growth and proliferation. In contrast, if the predominant interaction for creating a mesenchymal TME occurs between Notch and Jagged ligands, these cells tend to become more motile and invasive [297,298]. In addition, the ECM of tumour tissues provides a rigid scaffold for cells, which at the same time is a storage place for substances such as growth factors, chemokines, and cytokines. In particular, the ECM and TME are very important sources for TGFβ, which may—on the one hand—promote the differentiation of normal squamous epithelial cells; but on the other hand, may also potently promote EMT in transformed, squamous cancer cells. In part, this may also be achieved by inducing NOTCH1-target genes. Recent research has shown that the NOTCH1 intracellular domain (N1ICD) and TGFβ cooperate to enhance the expression of *SNAIL* and *ZEB1* in squamous cell carcinoma [270].

### 3.8. Regulation of EMT by Other Pathways in HNSCC

It was observed that the EGFR/PI3K/AKT signalling pathway promotes cell proliferation and invasion, which also involves EMT [85,299]. In line with this, inhibition of canonical NOTCH1 pathways by the presence of gain-of-function mutations detected in some HNSCC patients, such as *NOTCH1^V1754L^* and *NOTCH1^C1133Y^*, can activate the EGFR-PI3K-AKT pathway (see also Section 3). The PI3Kinase pathway may have some additional connections to Notch, and EMT-promoting factors, as outlined in the previous chapter. Studies have further shown that 3-phosphoinositide-dependent kinase 1 (PDK1) promotes EMT through activation of NOTCH1 signalling pathway in hypopharyngeal cancer cells (HSCC). PDK1 binds to the N1ICD, and prevented its ubiquitin-mediated degradation [300]. Likewise, in tongue cancers, NOTCH1 activation has been shown to lead to promotion of EMT. Meanwhile, overexpression of the NUMB protein, a known inhibitor of the Notch1/RBP-Jκ/PTEN/p-FAK axis, leads to the restoration of the epithelial-like phenotype of cells [301], and therefore a reversal of EMT-like processes (=MET). Interestingly, in vitro studies have shown that chronic NOTCH1 activation or overexpression in HNSCC cells results in a marked reduction in the expression level of several EMT markers. In contrast, overexpression of the JAG1 ligand led to a significant increase in only one mesenchymal marker; Grainyhead-like protein 2 homolog (*GRHL2)*, while *vimentin* and *fibronectin-1* showed a downward trend [88]. Research has further shown that Notch signalling is also linked to the cellular redox status in OSCC. Glutaredoxin 3 (GLRX3) maintains a low intracellular level of reactive oxygen species (ROS), that influences survival and metastasis of cancer cells. Knockdown of *GLRX3* leads to a decrease in *NOTCH* and *HES1* expression, resulting in reversal of EMT [302].

Additional links, or crosstalk, also exist between Notch and fibroblast growth factor receptor (FGFR) signalling. Upregulated Notch signalling in oral squamous cell carcinoma (OSCC) patients, for example, has been correlated with a more invasive phenotype, likely via increased *FGF1* expression in these tumour tissues [303]. This, in turn, may significantly contribute to cell migration and invasion via activating FGFR signalling. Not much is known about the precise links between Notch and FGFR signalling, tumour cell invasion and EMT or tumour cell plasticity in general. Additionally, expression of EGF, driven by an improved glycolytic metabolic programme in OSCC cells, has been shown to lead to metastasis—potentially by inducing EMT and stemness [304]. Last not least, co-staining of NOTCH1 and vascular endothelial growth factor (VEGF) in tongue cancer tissues by immunohistochemistry (IHC) showed a positive correlation between expression of these molecules, stemness, and cancer invasiveness [305].

There is also growing evidence concerning the putative links between Notch signalling, EMT, and various epigenetic processes. The role of EP300 in Notch downstream signalling has been outlined above (Figure 1 and Figure 2), also the involvement of the DNA demethylase LSD1. Strong inhibitory effects on EMT-like activities were observed after treating the TE9 oesophageal cancer cell line with valproic acid [306]. This is similar to results from other tumour entities in which EMT was also blocked after treatment with other HDAC inhibitors, such as Vorinostat [307]. Yet, surprisingly, there are only a few reports on any connections between epigenetic therapies and EMT, or involvement of Notch signalling as a potential mediator pathway. In preclinical models based on HNSCC and oral epithelial cell lines, a correlation between *SNAIL* expression, EMT-like activities and the EGFR-inhibitor Erlotinib resistance has been reported [308]. This is functionally similar to the connections observed between partial EMT, SLUG expression and transcriptional activities [309].

### 3.9. Notch, EMT and miRNAs

It is further noteworthy that exosomes, typically secreted by tumour or stromal cells, may contribute to drug resistance, mainly since they have a strong potential to modulate and re-direct tumour progression. How exactly exosomes may modulate tumour cell plasticity and invasiveness, however, is subject to intense ongoing research. One likely association relates to the presence of miRNAs in exosomes, and the potent effects of many miRNAs on regulating tumour cell gene expression. In HNSCC, tumour-derived exosomes have been shown to induce EMT and promote treatment resistance in the TME [310]. Specifically, these exosomes contain miR-155, a miRNA known to induce EMT-mediated development of drug resistance in several types of tumours. Other studies support similar mechanisms to occur in the context of HNSCC tumour progression, specifically with acquisition of Cisplatin resistance in oral cancers [311]. In yet another study, a different miRNA (miR-485-5p) was found to be associated with the inverse effect, namely the inhibition of EMT-like process in oral cancer cells. Exosomal transfer of miR-485-5p resulted in decreased *PAK1* protein expression in OSCC cells. This reverted EMT-specific motile cell properties, significantly inhibited invasion potential, and simultaneously re-sensitized Cisplatin-resistant cells for the drug [312]. Studies such as these probably only represent the tip of the iceberg, pointing to the immense potential of miRNAs to regulate dynamic processes such as tumour cell plasticity. This only adds to a large body of findings, relating members of the miR200 family to regulation of EMT and tumour cell plasticity.

Another important aspect in which tumour cell plasticity, partial/hybrid EMT, and enhanced stemness are involved is tumour dormancy. In vivo studies have shown that high expression levels of PRRX1, a member of the paired family of homeobox proteins that functions as a transcription co-activator, promoted cellular plasticity and tumour dormancy of HNSCC cells [313]. PRRX1 is itself partially regulated by miRNAs, especially miR-642b-3p. It was shown that overexpressed PRRX1 was closely correlated with downregulation of miR-642b-3p and led to increased tumour cell invasiveness and migration. Maybe most importantly, the interaction between PRRX1 and miR-642b-3p seems to regulate EMT status, and simultaneously help keeping malignant cells in a dormant state. In this fashion, partial EMT may also contribute to the poorly understood phenomenon of tumour dormancy in cancer, and specifically in HNSCC [313]. In which way exactly Notch signalling may be involved in these miRNA-regulated processes, and vice versa, remains almost completely unknown, but some studies have begun to explore this possibility more systematically. This has led to the identification panels of miRNAs and EMT-related “gene signatures” as potential biomarkers [140,314]. Several of these studies also systematically explored Notch pathway or signalling activity as one of the critical parameters.

### 3.10. Multiple Links between EMT; NOTCH Signalling, and Cancer Stem Cells/Stemness

Cells characterized by the E/M hybrid phenotype not only strongly promote their capacity of collective epithelial migration, for example in the form of multicellular clusters or organoid-like cell aggregates. These cell clusters not only have a higher chance of survival in the blood stream as circulating tumour cells than single cells; they may also show an increased potential for extravasation and forming distant micro-metastases [247,251]. Such collectively migrating cancer cell aggregates may represent a pivotal role in formation of distant metastasis. Their presence in the circulating tumour cell population isolated from HNSCC cancer patients correlates with poor prognosis [253,315,316]. In addition to the E/M phenotype, it is likely that an enhanced cancer stem cell (CSC) phenotype, or increased stemness, contributes to such observations, and that Notch signalling may play its part in these processes. For example, it was shown that *NOTCH1* expression was not only related to poor patient outcome, but also that knockdown of *NOTCH1* induces potent antitumour effects in an in vivo model, related to HNSCC stem cells, or CSCs [279]. Earlier studies had reported and validated that activation of NOTCH1 signalling is related to enhanced tumour stemness, or formation of overt CSCs, which can be isolated from HNSCC patients. This process is likely associated with a crosstalk between the Notch and Wnt signalling pathways [317,318]. In addition, Chang and Lei aimed to identify driver genes related to the Notch–Hedgehog pathways by bioinformatic analyses based on data mining in the *Kyoto Encyclopedia of Genes and Genomes* (KEGG) database. They were able to link multiple candidate genes identified in this exercise to tumour stemness, hypoxic conditions, and various immune components. This network includes NOTCH1 itself, which was found to be a significant prognostic gene for HNSCC patients [319]. Upadhyay et al. showed that *NOTCH1* overexpression in HNSCC cell lines facilitates spheroid formation, which were markedly enriched for stem cell characteristics [320]. In contrast, knockdown of *NOTCH1* expression led to the reverse effects—impaired spheroid formation, indicating *NOTCH1* as a central stimulator and regulator of (tumour) cell stemness. Moreover, inhibition of *NOTCH1* in HNSCC was found to enhance the efficacy of chemotherapeutics targeting the largely elusive pool of stem cells [111].

*NOTCH1* was also found to be crucial for the maintenance of stemness in oral squamous cell carcinoma (OSCC) [321]. This study indicated that the Notch pathway can be activated by prolonged exposure to tumour necrosis factor alpha (TNFα), a cytokine with potent proinflammatory effects, which overall stimulated the selection and maintenance of stem cell like-properties. Contrary to the NOTCH receptors, little is known about the roles of Notch ligands in regulating tumour stemness and maintaining CSCs in HNSCC. So far, only JAG1 was found to be upregulated in this type of cancer cells, and also correlated with poor prognosis [298]. Bocci et al. presented a computational model of E/M, and predicted CSCs that displayed an enhanced Notch-Jagged signalling [322]. Although this virtual model was created based on CSCs derived from various cell lines, the CSC of HNSCCs were not included in the study. It would be of great interest to investigate whether the computational model also applies to HNSCC. In their subsequent in vitro studies, the same authors were able to show that Notch is a key driving force for the maintenance and heterogeneity of CSCs. In short, they showed by knocking down JAG1 expression in the breast cancer cell line SUM149 had a serious impact on organoid formation [323], which they assume is due to reduced stemness. These studies also point towards CSCs as a potent mechanism that provides the ability to metastasize to tumour cells. Active NOTCH4 signalling was also reported to induce EMT in HNSCC [115,281]. Additional, very detailed studies were performed to further investigate the role of NOTCH4 in this context [315], also in other cancer entities. We strongly recommend two recent reviews, which cover the topic of epithelial–mesenchymal plasticity, TME and their contribution to developing CSC [260,316]. The alternatives for targeting the Notch signalling pathway in CSCs are summarized in a review by Venkatash et al. [324]. The authors tried to collect the list of GSIs and monoclonal antibodies under investigation, with the goal to block Notch activity in tumour cells, including CSCs.

As a summary, the continuum of cell-plasticity states, including stemness, significantly complicates (a) the emergence of a pronounced intra-tumour heterogeneity in HNSCC, (b) the variable (and poorly understood) role of cancer stem cells (CSCs) within the cancer tissue, (c) the elusive nature of the stem cell niches, and d) the maintenance of CSCs during and after chemo- or radiotherapies (tumour dormancy). All these aspects may be related to locoregional recurrence or relapse in HNSCC, although full experimental proof is usually missing. Especially CSCs and tumour dormancy, just as the potential involvement of Notch signalling in these processes, remain poorly understood concepts in HNSCC. This is mainly because appropriate in vitro model systems are still largely missing that allow functional validation of specific scientific concepts, targets, mechanisms, and pathways—in the context of a living tissue. Nevertheless, some of these aspects have been traditionally addressed in mouse models (and thus living tissues), which represent a strong restriction due to high cost, ethical considerations, low experimental throughput, also because of significant differences between mouse and human genetics and cell biology. In addition, slow progress in understanding complex cell and tissue biology relates to the fact that cellular processes within living (cancer) tissues—regardless of human or mouse origin—are still essentially a “black box” and cannot be easily monitored and quantitated. Intravital monitoring of tumour cell behaviour in the context of complex tissues (xenografts) is possible, but does not represent a widely used, mainstream technology. 

Alternative hypotheses, concerning the connection between NOTCH1 and cancer stem cell biology have also been formulated (reviewed in detail in Shah et al, 2020 [88]). Physiological Notch signalling in normal epithelial tissues (mucosae) may primarily allow for expansion and persistence of stem cells. These can accumulate additional genomic alterations, which will eventually provide them with additional growth advantages. This normal physiological function may relate to the observation that high levels of Notch proteins in cancer cells confer relative resistance to chemotherapies, and enrichment of cancer stem-cell like populations. If this is correct, Notch signalling would primarily activate genes mediating early, lineage-specific differentiation decisions. Hijacking this process by cancer cells would transform tumour cells into a transiently amplifying, rapidly proliferating phenotype, that may also include pro-survival and anti-apoptotic signalling. This would be consistent with parallel observations that NOTCH1 activation increases resistance to anoikis.

## 4. Targeting Tumour Angiogenesis in HNSCC

Neo-angiogenesis is a physiological process that includes the formation of new blood vessels from pre-existing blood vessels, followed by penetration and growth in the tumour ecosystem or microenvironment. Solid cancers, and specifically carcinomas such as squamous cell carcinomas (SCC), depend on angiogenesis to grow and metastasize. Emerging cancer tissues are not able to grow beyond a size of approximately 1 mm in diameter, without effectively triggering the formation of new blood vessels or capillaries. They later also require blood and lymph vessels to migrate to places far away from their place of origin. Angiogenesis is typically induced by hypoxia, or low oxygen levels, prominent in transformed or pre-malignant tissues. In response to hypoxia, cancer cells or CAFs start to secrete pro-angiogenic signals to stimulate blood vessels. These, in turn, will help tumour cells by creating new extensions. Several potent signalling cascades can activate angiogenesis, triggered by growth factors such as Vascular endothelial growth factor (VEGF), Platelet-derived growth factor (PDGF), Basic fibroblast growth factor (bFGF), Transforming growth factor beta (TGFB1), EphrinB2, Tumour necrosis factor (TNF), Epidermal growth factor (EGF), or Interleukin-8 (IL-8). On the other hand, some elements play an inhibitory role in angiogenesis, such as Angiostatin (see below), Interferons, Platelet factor 4 (PF4), Thrombospondin-1 protein (THBS1), Prolactin, or Interleukin-12 (IL-12). The balance between activating and inhibitory factors is crucial for the spread and progress of angiogenesis. Tumour angiogenesis primarily requires the recruitment of the vasculature by inducing the sprouting of existing vessels/capillaries, to supply the tumour tissue with nutrients. Sprouting angiogenesis requires tight regulation between endothelial tip and stalk cells; and Notch signalling is a basic component within this process. Basically, tip cells (with high DLL4 levels) differentiate in response to pro-angiogenic factors, to establish new vessels. In contrast, Notch-mediated VEGFR2 inhibition sustains the endothelial stalk cell phenotype, thus preventing further sprouting, and controlling the vasculature architecture [325,326]. Other signals that have very significant effects on angiogenesis are counteracting the Notch ligands JAG1 and DLL4. We will see that these ligands are particularly potent in HNSCC, and also show differential effects.

Tip cells with high levels of DLL4 activate NOTCH1 in stalk cells, elevate NOTCH signalling and locally induce differential gene expression. Enhancement of DLL4 on tip cells is achieved by both endothelial and non-endothelial derived VEGF, and by activation of VEGFR2 [327]. Meanwhile, in the stalk cells, activation of the same receptor results in release of the NICD and regulation of expression of target genes such as *PTEN*, inhibiting proliferation. Simultaneously, the NICD induces targets such as Mothers against Decapentaplegic Homolog (*SMAD6*), which counteracts and locally titrates the signalling mediated by Bone morphogenetic proteins (BMPs). Regulation of new sprouting during vascular expansion in cancer tissues is therefore based on the tight integration of BMP, NOTCH, and VEGF signalling [328]. Synchronized fluctuations of these pathways support vessel enlargement and negatively regulate branching. In adult vessels, NOTCH signalling is in charge of maintaining endothelial quiescence and junctional integrity [32].

### 4.1. (Neo-)Angiogenesis in HNSCC

Only relatively few studies have investigated the Notch-mediated regulation of angiogenesis in HNSCCs; many of these studies date back decades. There are relatively few recent studies, which we will outline below. The role of Notch signalling in angiogenesis in HNSCC is closely related to the functions of the VEGF receptor (VEGFR) and its ligand, VEGF. Like in many other cancer types, hypoxia potently induces VEGF expression by hypoxia-inducible factor (HIF-1α) in HNSCC tissues [329]. It was further shown that mitogen-activating protein kinase (MAPK) induces JAG1 protein, followed by expression and trans-activation of Notch signalling in neighbouring endothelial cells (ECs), which leads to the promotion of capillary-like angiogenic sprout formation [293]. Both, endothelial DLL4 [35,330,331] and JAG1 (overexpressed via MAPK pathways) [293,332], are known to promote angiogenesis in the TME via NOTCH1 activation. The expression of endothelial JAG1 or 2 in HNSCC tissues has been observed for a long time [332], especially in endothelial cells within the tissues. The most intensely investigated mechanisms of Notch-mediated angiogenesis in HNSCC relate to the complex biology of the ligands DLL4 and JAG1 or JAG2; and these factors have also been identified as prognostic factors in HNSCC patients [31,33,298], or as potential pharmaceutical targets [32].

Another likely executor of these regulatory mechanisms in neo-vascularization active in HNSCC is the Angiopoietin system. The secreted factors or tissue hormones Angiopoietin-1 (Ang-1) and Angiopoietin-2 (Ang-2) show potent effects on tumour angiogenesis and are in turn modulated by Notch signalling. Notch signalling mediates VEGF and Ang-2-induced cell tube formation. Furthermore, Ang-1 has the potential to decrease endothelial cell permeability and vascular stabilization, specifically via the recruitment of pericytes and smooth muscle cells to growing blood vessels. In contrast, Ang-2 mainly mediates angiogenic sprouting and vascular regression. This antagonism between Ang-1 and Ang-2 thus affects perivascular cell coverage in new vessels. In the presence of low VEGF levels, vessels typically regress. In the presence of VEGF, however, the endothelium becomes activated, and vessels proliferate. VEGF levels are thus crucial also for the effect of the antagonistic pathway [333].

### 4.2. The Jagged1/Notch Signalling Axis in Angiogenesis

The selective choice of interaction between NOTCH-DLL4 over NOTCH-Jagged1 is not only dependent on the respective protein expression levels, but also controlled by a set of Fringe proteins (O-fucose-beta1,3-N-acetylglucosaminyltransferases). The expression of three essential mammalian fringe proteins (Lunatic (LFng), Manic (MFng), or Radical (RFng) Fringe) was shown to enhance DLL1 binding and activation of Notch1 signalling in HEK293T and NIH 3T3 cells. It was further observed that LFng and MFng proteins suppressed Jagged1-induced signalling, and DLL1 or JAG1 ligands were able to induce RFng [334]. The mechanisms of O-glycosylation by Fringe proteins on NOTCH receptor interactions are still under intense investigation, as recently reviewed by [335]. Additionally, transformed squamous carcinoma cells can express significant amounts of JAG1 and 2 proteins, the role of which remains unclear: are these able to trigger Notch-signalling via auto-stimulation, by cis-acting cell–cell-interactions? Alternatively, are they attenuating Notch signalling by cis-inhibition?

Pedrosa et al. investigated the effects of endothelial JAG1 ligand expression on HNSCC progression. Overexpression of JAG1 in endothelial cells increased the growth of subcutaneous Lewis lung carcinoma (LLC) tumour transplants [332]. The mechanisms how endothelial Jagged1 may functions as a pro-angiogenic ligand in tumours has been investigated in depth. In a first step, Jagged1 is again antagonizing with DLL4, impedes DLL4/NOTCH1 activation, and blocks its regulation of endothelial branching. This, in turn, positively regulates *VEGFR2* transcription, but negatively affects *VEGFR*1 transcription. Endothelial Jagged1 also has the capability to positively control the growth and proliferation of Vascular smooth muscle cells (vSMC). This appears to be mediated by interfering of Jagged1 with the activities and expression levels of NOTCH3, and the Notch-target gene hairy/enhancer-of-split related with YRPW motif-like (HeyL). This is in accordance with our current understanding, namely that activation of perivascular NOTCH3 and the HeyL effector is essential for the assembly of a submucosa layer (SM); and critical for vascular integrity and functionality. Last not least, endothelial Jagged1 also strengthened tumour dysplasia via two different effects: (1) a pro-angiogenic effect, enhancing tumour vascular density, maturation, and perfusion, and (2) an angiocrine outcome, likely through stimulation of NOTCH3/Hey1 and its effects on tumour cell proliferation. Based on these data, it appears feasible that Jagged1 represents a potential target for therapies [32,332,336], quite in contrast to DLL4, which shows partially controversial effects [295,330,337,338]. Nevertheless, DLL ligands remain in the interest of pharmaceutical developments as a potential drug target [35].

### 4.3. The DLL4/NOTCH Signalling Axis in Angiogenesis

Blocking the pro-angiogenic activities of the DLL4-NOTCH axis may be an interesting strategy for targeted therapies [35]. In a non-pathological state, after activation, the proangiogenic effect of DLL4-NOTCH is reduced by a negative feedback loop associated with a decrease in VEGFR expression. This consequently leads to reduced sensitivity of the cells to VEGF [338,339]. However, it was shown that prolonged artificial inhibition of DLL4 causes pathological activation of endothelial cells, leading to impaired blood vessel morphology and histopathological changes in organs [340,341]. This is followed by upregulation of the VEGF2 receptor in endothelial cells, which in turn increases their sensitivity to VEGF and supports angiogenesis [340]. Additionally, Djokovic et al. show that in the early stages of carcinogenesis, a lower DLL4 level is associated with vascularization in the TME, again through increased VEGFR2 level [342]. Therefore, blocking DLL4 in the early stages of cancer development may be risky. Specifically, Djokovic et al. investigated the consequences of gene dosage, or partial DLL4/Notch signalling [342] on squamous cancers, specifically skin papillomas. The results showed that partial DLL4/Notch inhibition by haploid deletion of DLL4 in papillomas (DLL4^+/−^) promotes the formation of less mature, but nevertheless productive angiogenesis in skin papillomas. These pre-malignant tumours showed a ~35% increase in vessel density, however, with typically disorganized endothelial networks. The immature nature of these capillaries is further indicated by incomplete, flawed branching and thin, non-functional interconnections. A reduction of DLL4 dosage by 50% permitted capable neo-vessel formation. This simultaneously promoted a marked increase in papilloma growth and corresponding mouse models [338,342]. In contrast, DLL4 overexpression improves vascular maturation and functionality in the mouse model, resulting in increased vessel dimensions, and network perfusion [338,339]. The mechanism behind the DLL4/Notch interaction in cancer tissues likely relates to the ability of this heterotypic signal to select endothelial cell departure from the pre-existing, activated endothelium, and to organize new sprout-like outgrowth that is induced by an altered VEGFR2/VEGFR1 ratio (as outlined above).

The function of the DLL4/Notch axis has been further investigated in very elaborate studies, utilizing diverse truncated Notch proteins, or protein domains. Klose et al. aimed to observe how very small soluble ligand or receptor domains, namely the DSL domains of the ligands DLL1, DLL4, or JAG1 and the EGF-like repeats 11–13 of NOTCH1, may affect signalling outcome and vascular differentiation [343]. Again, increased DLL4-Notch signalling potently repressed vascular branching and outgrowth. Simultaneously, blocking of this signalling can result in increased branching and outgrowth. For example, a panel of neutralizing antibodies, soluble DLL4 ligands, or GSI were shown to effectively modulate the DLL4/Notch-related inhibition of Notch signalling. In particular, truncated ligand proteins, such as DLL1-Fc, DLL4-Fc, and JAG1-Fc proteins, effectively impede the Notch signalling pathway in both 2D and 3D cell cultures, regardless if these proteins possess the complete extracellular domain or only the DSL domain [343]. Soluble, truncated receptor fragments such as the NOTCH1-EGF 11-13-Fc protein have a more complex function in angiogenesis, which strongly depends on different conditions between 2D or 3D cell culture. These effects also strongly depend on cell- and tissue culture conditions, and the nature of cell–cell-interactions. In 2D monolayer cell cultures, in which cells interact only on superficial level, Notch signalling is hindered by the fragmented proteins. In contrast, signalling activity markedly was elevated in differentiation-promoting 3D cultures, and angiogenic sprouting was significantly disturbed. In addition, the complete NOTCH1 extracellular domain fused to Fc (resulting in a NOTCH1 decoy) strongly impeded primary endothelial cell cultures (HUVEC) sprouting and even inhibited tumour angiogenesis in mice. Thus, Delta-like and Jagged ligands bind to NOTCH1 with different affinities, and NOTCH1 generally has a stronger binding affinity to the inhibitory ligands JAG1 and 2. Application of DLL1-DSL-Fc (Delta like protein 1- Delta/Serrate/lag-2, DLL4-DSL-Fc, and Jag1-DSL) proteins generated extreme vessel branching and increased tip cell formation in the neonatal mouse retina. In contrast, the application of NOTCH1-EGF 11-13-Fc inhibited tip cell formation. The resulting vascular pattern showed significantly less branching and complexity. These are the classic indications of the Notch barricade, whereas application of NOTCH1-EGF11-13-Fc prevented tip cell formation and vessel branching. These data are obtained from JAG1 overexpressing mice, Notch1 gain-of-function mice as well as tumour studies using the Notch1-decoy [325,343].

### 4.4. The Extracellular Matrix, Notch Signalling, and Angiogenesis

During active (neo-)angiogenesis, endothelial cells secrete different types of extracellular matrix proteins that produce a planar protein network. This planar protein network enables a significant barrier function and effectively encapsulates blood within the capillaries, and is known as basement membrane (BM), or basal lamina. The BM serves as a 50–200 nm thick, static, and planar protein network that provides a dynamic extracellular environment crucial for blood vessel integrity. The BM largely consists of macromolecular collagen IV (Col IV), laminins (4-1-1 and 5-1-1), perlecan, fibronectin, and nidogen which together from a semi-rigid ECM, secreted by endothelial cells and (probably) supportive pericytes. Gross et al. found that collagen type IV (COL4) secretion and deposition in angiogenesis by the modulation of trafficking mediators is functionally linked with permissive Notch signalling. Basically, the process starts with the activation of the Rab10 intracellular vesicle transport protein, mediated by Notch downstream nuclear signalling. Rab10 then works in combination with another vesicle transport regulator, Rab25, to deliver Lysyl hydroxylase 3 (LH3) to Col IV-containing vesicles (CIVC) for secretion. By blocking Rab10 or Rab25 expression and function, transportation or trafficking of LH3 is prevented, and Col IV secretion in endothelial cells is drastically reduced. Apart from Rab10, also transcription of guanine nucleotide exchange factor (GEF) and DENN domain-containing protein 4C (DENNd4C) are activated by Notch signalling and affect LH3 trafficking and activity. In endothelial tip cells with low Notch levels, VEGFR2 expression is elevated, leading to the reduction of Col-IV secretion to deviate energy to migration and ECM degradation. On the other hand, stalk cells with a high Notch level contribute to the newly made vascular tunnel by secreting Col IV with the help of activated LH3 trafficking [344].

Another important factor that may affect angiogenesis in HNSCC is vimentin, one of the “classic” mesenchymal biomarker proteins that are often induced by an EMT in squamous carcinoma cells. This intermediate filament protein physically interacts with the ligands Jagged1+2, and mediates or promotes Notch activation upon receptor binding [345]. In addition, reduction of vimentin protein expression in epithelial tissues limits Jagged-mediated transactivation and increases the expression of the modulatory Fringe proteins mentioned above, with the potential consequences of disrupting sprouting angiogenesis. Vimentin further enhances endothelial cell invasion by strengthening the complex between the scaffolding protein Receptor of activated protein C kinase 1 (RACK1) and focal adhesion kinase (FAK), which is utilized to control FAK activity. FAK itself is closely linked to integrin signalling and represents one of the major execution pathways, defining how integrins control the interactions between tumour and endothelial cells, the stroma, and the ECM. Vimentin also promotes endothelial cell invasion in 3D collagen matrices, by complexing with and stimulating membrane translocation of the metalloproteinase MT1-MMP, which is responsible for completed sprouting responses [345]. Estrach et al. showed that the central, laminin-binding integrins α2β1 and α6β1 function as potent upstream regulators of Notch pathway activity, and that VEGF stimulation of EC enhances laminin production and DLL4 expression [346]. As a result, and mediated by VEGFR and Notch signalling, VEGF supports laminin production and deposition by ECs. In turn, specific production of laminin activates integrin-dependent signalling, which induces DLL4 expression and Notch pathway activation. Thus, a highly complex regulatory cascade, linking VEGF, integrin, and Notch signalling, appears responsible for the regulation of tip cell/stalk cell balance in the vasculature [346].

**Figure 4:**
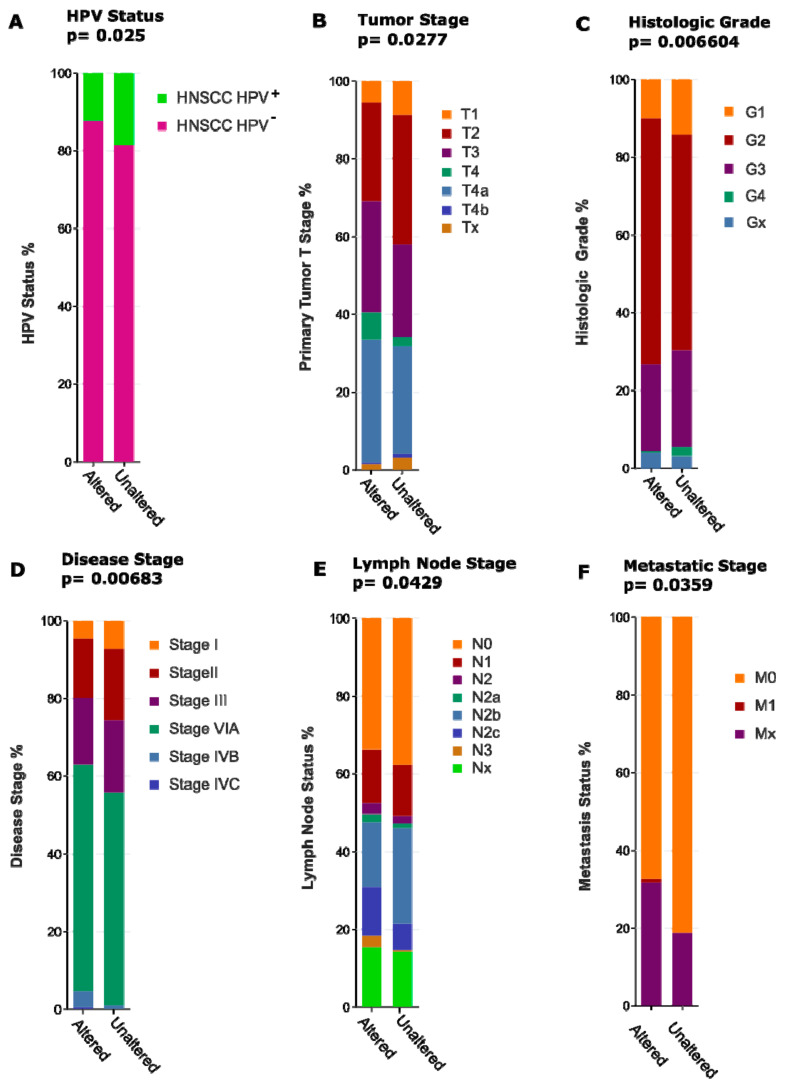
Mutation status of most relevant Notch-pathway related genes incl. *NOTCH* receptors correlates with various clinical and pathological parameters in HNSCC patients and tissues. Tumours with mutations in prominent Notch pathway-related genes (“altered group”) are less frequently HPV^+^ (**A**). Patients with Notch-pathway alterations present with significantly advanced tumour stage (**B**), histologic grade (**C**), and disease stage (**D**). These patients also more frequently develop lymph node metastases (**E**) and distant metastases (**F**).

**Figure 5:**
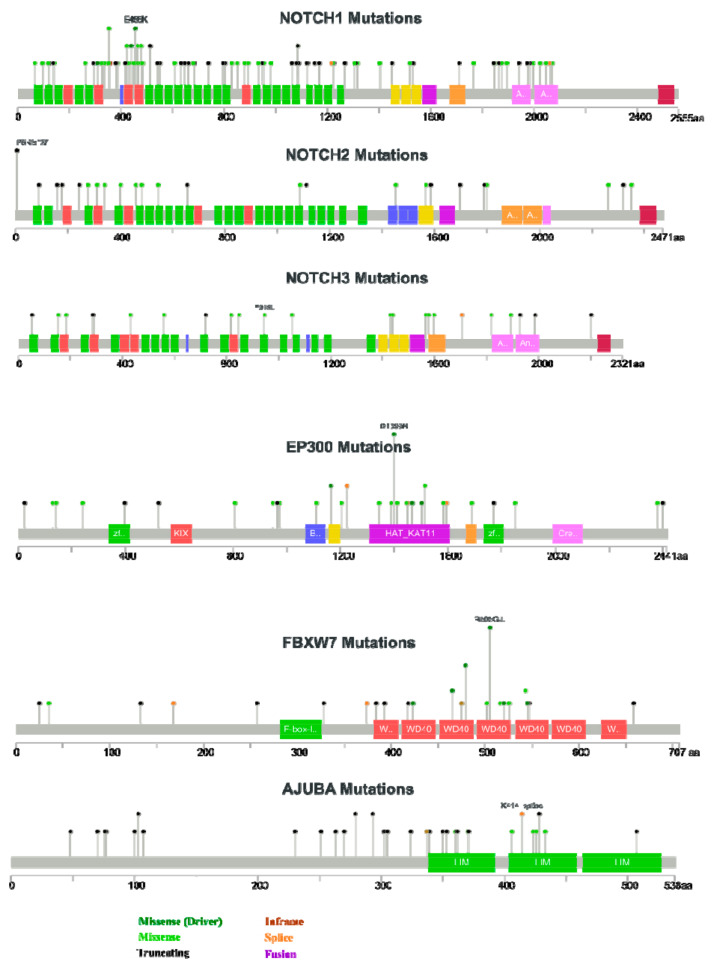
Lack of mutation hotspots in Notch pathway-specific target genes *NOTCH1*-*3*, Histone Acetyltransferase P300 (*EP300*), LIM Domain-Containing protein Ajuba (*AJUBA*), and F-Box and WD Repeat Domain Containing protein 7 (*FBXW7*). NOTCH receptor mutations are evenly spread across the entire length of extracellular protein domains, likely resulting in loss-of-function mutations and truncated proteins. Mutations in other Notch-related target genes such as *EP300* and *AJUBA* also do not show any prominent, recurrent hotspots, but can be focused on functional domains, such as the HAT/histone acetylation domain of *EP300*.

**Figure 6:**
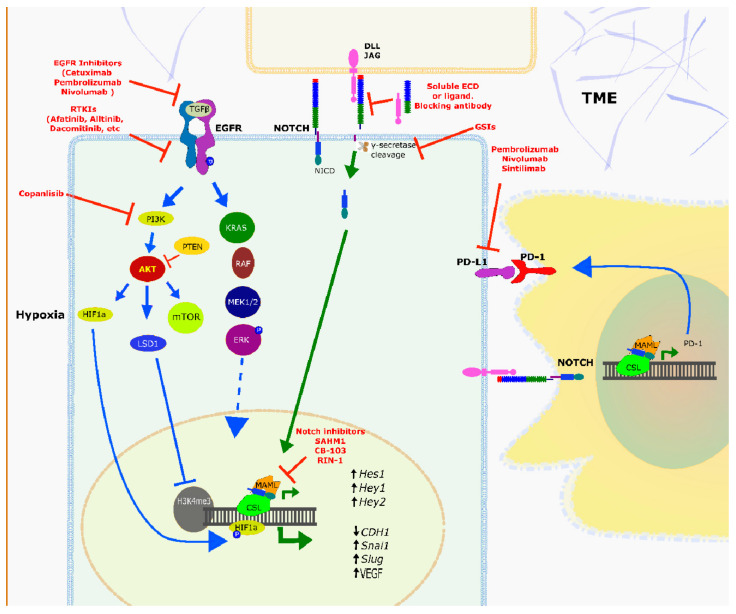
Schematic summary of pathways, and targeted drugs, as described in chapter 2, with a special focus on mechanisms that involve Notch signalling.

**Figure 7:**
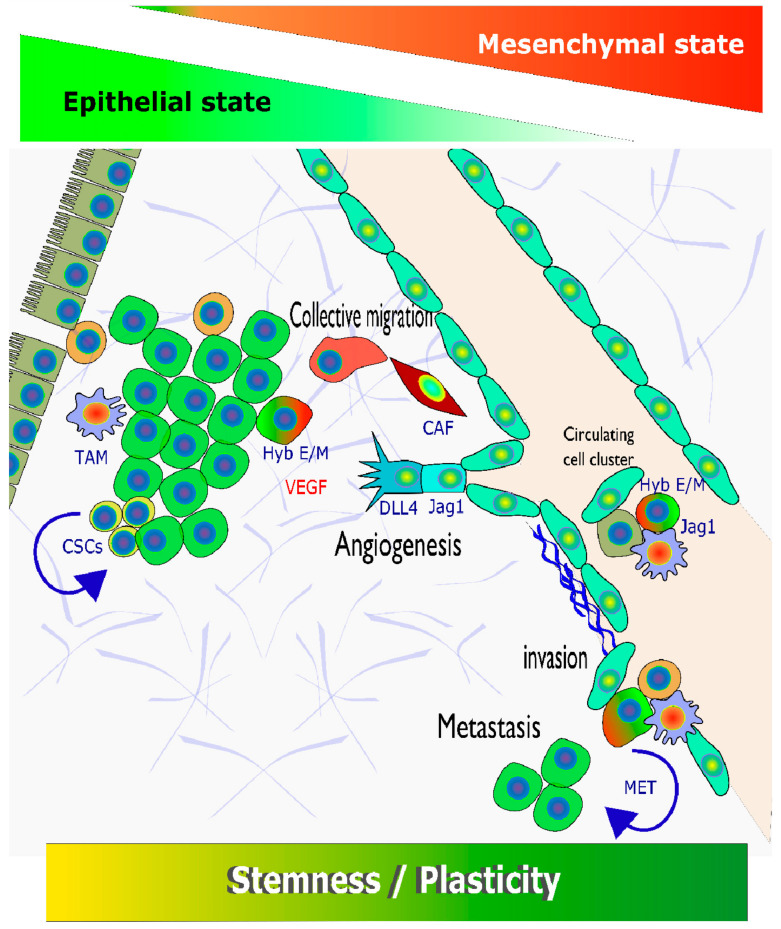
Spectrum, or phenotypic continuum of dynamic, cancer-relevant processes promoted by Notch signalling that result in various aspects of tumour cell plasticity. The transgression between epithelial and mesenchymal states, also towards increased stemness, represents a dynamic process that is influenced by a plethora of internal (genetic, epigenetic) and external (ECM, TME, growth actors, and cytokines) factors.3.3. Specific Features and Drivers of EMT in Squamous Cell Carcinomas.

Another process likely affecting angiogenesis in HNSCC is the HPV status. In HPV^−^ head and neck cancers, angiogenesis appears mainly driven by independent effects of NOTCH1 and EGFR, and their impact on HSNCC progression appears complementary. Furthermore, HPV^−^ cancers possess higher growth or proliferative potential, metastasize more frequently, and respond less well to therapies [13]. On the other hand, HPV^+^ head and neck cancers do not show concrete correlation with Notch pathway activities in angiogenesis, and are also less dependent on EGFR signalling [13,347]. There are also striking differences between the tumour (and immune) microenvironment of HPV^+^ and HPV^+^ cancers [26,348] that may affect response of patients to immune checkpoint inhibitors [23,25], and the formation of an immune-refractory TME. These tumours generally have lower growth and proliferation potential, compared to HPV-negative cancer cells. The possible mechanism that drives angiogenesis in HPV^+^ tumours are not precisely understood as of yet [349], but Notch signalling may be involved in these processes. However, it is evident that the frequency of NOTCH1 mutations and Notch pathway activation is different in HPV^−^ and HPV^+^ tumours (Figure 4A).

## 5. Discussion—Future Outlook

We are only beginning to understand the nature of Notch signalling in HNSCC. Particularly, the bimodal activity of Notch receptors to act as both tumour suppressors, and proto-oncogenes—maybe only at later stages of cancer progression—is currently not fully elucidated. Notch pathway activities, although initially acting as tumour suppressor, may have the built-in potential of recovery or reconstitution, even hyperactivity. This may specifically apply for advanced HNSCC, such as relapsed/metastasizing tumours (R/M HNSCC), and there is overwhelming evidence that hyper-activity of Notch signalling clearly does correlate with decreased overall, or disease-free survival [109,350,351]. If we want to better understand this mysterious conversion, we need to address the intrinsic complexity of the Notch pathway as a whole: which roles do the diverse receptors and ligands play? How does signalling proceed, even in the presence of mutations in one of the receptors? Why are there not more gain-of-function mutations in NOTCH1-4 receptors in HNSCC, when the Notch pathway itself appears to be activated by many tumours, especially in late-stage tumours? Additionally, how is it possible that loss-of-functions in one of four receptors (most frequently, NOTCH1), has the potential to effectively silence the entire pathway, while continued expression of other receptor variants is recorded? Where do individual, downstream signalling cascades, regulated by each one of the four receptors overlap or synergize, and where do they differ? In this context, it also remains rather puzzling why NOTCH1 mutations are so strikingly pre-dominant in HNSCC (found in 17% of tumours), despite significant expression levels observed for the other three receptors, in the same tissues and even the same cells. NOTCH2 and 3 are frequently upregulated in some of these lesions [43,109,352], including some that show NOTCH1 mutations; and have been associated with poor patient outcome. It may therefore be necessary to better differentiate between the differential and (potentially) unique roles of the four NOTCH receptors themselves and identify the mechanisms how precisely mutations in usually only one receptor can be overcome later. Does this occur by gain-of-function modifications of Notch proteins, by over-expression or mutation of Notch interacting molecules, such as the ligands—or by modulating downstream activities? Is hyper-activation and recovery of a once shut-down pathway led by one of the other receptors, or by amplifications and overexpression of one of the key Notch-response genes such as *HES1*, *Cyclin D1*, *c-Myc*, or others [281,353], which is very frequently observed.

The precise roles of cis- and trans-activation of NOTCH receptors by the ligands also remains a riddle. Additionally, the Notch ligands JAG1/2 and DLL1/3/4 are rarely affected by deletions or incapacitating mutations, and instead, are often found over-expressed [31,32], for example in tongue cancers. Why would Notch ligands be continuously expressed or even over-expressed in cancer cells (not only the stromal or endothelial cells), if not for the fact that the Notch signalling pathway is still functional or even hyperactive in these cancers. The role of Notch signalling in HNSCC thus remains interesting, although the focus of attention for drug discovery and target identification/validation may need to move forward a few steps and include upstream and downstream Notch pathway executioners and mediators. A concerted effort may also need to focus more on other cell types than only the transformed, squamous epithelial cells located in the TME.

As an alternative to investigating the precise role of all four NOTCH receptors, or the five ligands in cancer cells, would be the use of functional tests. These could be measuring and integrate net Notch pathway activity. Such integrated approaches may be more reliable and straightforward, especially for considering clinical decision-making or stratification, and identifying patients that may benefit from targeted therapies [42]. Such a test can be based, as described, on measuring the expression level of a Notch-related gene signature (including the usually suspects such as *Hes1* and *Hey1*, etc.), combined with evaluating intracellular NICD levels. Such tests could then lead to a generalized “Notch activity score” that can be correlated with clinical aspects, such as shorter event-free or overall survival in patients showing higher Notch activity. Useful, generalized “Notch overview” tests would also integrate all possible receptors and ligand/receptor activities, and eliminate the need to discriminate between the many different types of mutations found by genetic analyses (PCR combined with directed sequencing). Similar approaches, measuring the presence of the cleaved NOTCH1 NICD in tumour cells or tissues, have been suggested earlier [354], combined with or alternative to measuring the overexpression of *Hes1* [351].

In short, determining the precise role and activity of NOTCH receptors plus the potential hyperactivation of Notch pathway activities may become critical for determining a patient’s response to therapies (=personalized medicine), and predicting the individual risks for tumour progression, relapse and metastasis. Such tools may also lead the way towards future early stage drug discovery programs related to Notch signalling, and identification of novel Notch-related molecular targets.

## Figures and Tables

**Table 1:** Ongoing or recently completed clinical trials in HNSCC exploring experimental therapies.

Intervention/Treatment	Molecular Target	ClinicalTrials.gov Identifier
Alisertib (MLN8237)	Aurora A kinase	NCT01045421
Axitinib (AG-013736)	VEGFR	NCT01469546, NCT02762513
	EGFR; tubulin; DNA	NCT00736944
Cetuximab + Abraxane (protein-bound Paclitaxel) + Cisplatin + 5-FU + Radiation	EGFR; DNA	NCT01218048
Cetuximab + Cisplatin or carboplatin + Surgery + Post-surgical radiation	EGFR	NCT01012258
Cetuximab + concomitant boost radiotherapy	EGFR; nucleic acids	NCT01581970
Cetuximab + Cyclophosphamide	EGFR; tubulin; DNA	NCT01437449
Cetuximab + Docetaxel + Cisplatin + Carboplatin	EGFR; TLR9	NCT01040832
Cetuximab + EMD 1201081	EGFR; IGF-1R	NCT00957853
Cetuximab + IMC-A12 + Surgical tumour resection	EGFR; tubulin	NCT01412229
Cetuximab + Nab-paclitaxel + Carboplatin	EGFR; PD-1; DNA	NCT03349710
Cetuximab + Nivolumab + Cisplatin + Radiotherapy	EGFR; PARP1	NCT01758731
Cetuximab + Olaparib + Radiation Therapy	EGFR; tubulin; DNA	NCT02124707
Cetuximab + Paclitaxel + Carboplatin	EGFR; tubulin; DNA	NCT01566435
Cetuximab + Paclitaxel albumin-stabilized nanoparticle + Cisplatin + 5-FU + Intensity modulated radiation	EGFR;	NCT00768664
Dacomitinib (PF-00299804)	ALK1	NCT01458392
Dalantercept	PDK; DNA	NCT01386632
DCA (dichloroacetate) + Cisplatin + Radiation	PD-L1	NCT02207530
Durvalumab (MEDI4736)	PD-L1; CTLA4	NCT02319044
Durvalumab (MEDI4736) + Tremelimumab	PD-L1; CTLA4	NCT02369874
Durvalumab (MEDI4736) + Tremelimumab + Standard of Care	EGFR; DNA	NCT00304278
Erlotinib (Tarceva) + Intra-arterial Cisplatin + Radiation Therapy	immune system	NCT02163057
INO-3112 (DNA Vaccine)	immune system; DNA; COX1/2	NCT00210470
IRX-2 + Cyclophosphamide + Indomethacin + Zinc + Omeprazole	KSP	NCT00095628
Ispinesib	EGFR; DNA	NCT01044433
Lapatinib ditosylate + Capecitabine (5-FU precursor)	Eg5; immune system	NCT01059643
Litronesib (Y2523355) + Pegfilgrastim	Respiratory complex I; DNA	NCT02325401
Metformin + Cisplatin + Radiation Therapy	TLR8; DNA	NCT01836029
Motolimod (VTX-2337) + Carboplatin + Cisplatin + 5-FU	tubulin; EGFR; DNA	NCT00343083
Paclitaxel + Erbitux + Carboplatin + Radiation	CDKs; DNA	NCT03194373
Palbociclib + Carboplatin (5-FU prodrug)	EGFR	NCT00446446
Panitumumab	EGFR; DNA	NCT00756444
Panitumumab + Cisplatin + 5-FU	EGFR; DNA; tubulin	NCT00454779
Panitumumab + Cisplatin + Docetaxel	PD-1	NCT02255097
Pembrolizumab	PD-1; BTK	NCT02454179
Pembrolizumab + Acalabrutinib	PD-1	NCT03057613
Pembrolizumab + Radiation: IMRT 60-66Gy	PD-1; immune system	NCT02626000
Pembrolizumab + Talimogene laherparepvec	HDAC	NCT00084682
Romidepsin	immune system	NCT02013050
SGX942	EGFR	NCT01417936
Sym004 (Futuximab and Modotuximab)	PDE5	NCT01697800
Tadalafil	DNA; tubulin	NCT00270790
Taxol (Paclitaxel) + Carboplatin + Amifostine + Radiotherapy	mTOR; tubulin; DNA	NCT01016769
Temsirolimus + Weekly Paclitaxel + Carboplatin	4-1BB; MS4A1	NCT01307267
Utomilumab (PF-05082566) + Rituximab	tubulin; DHFR	NCT02347332
Vinflunine + Methotrexate		

## Data Availability

Publicly available datasets were analysed in this study. This data can be found here: https://www.cbioportal.org/study?id=hnsc_tcga (accessed on 17 November 2021).
